# Risk Factors for Recurrence After Surgery for Rectal Cancer in a Modern, Nationwide Population-Based Cohort

**DOI:** 10.1245/s10434-024-15552-x

**Published:** 2024-06-09

**Authors:** Sepehr Doroudian, Erik Osterman, Bengt Glimelius

**Affiliations:** 1 Department of Immunology, Genetics and Pathology, Uppsala University, Uppsala, Sweden; 2 Center for Research and Development, Uppsala University/Region Gävleborg, Gävle, Sweden; 3 Department of Surgery, Gävle County Hospital, Gävle, Sweden; 4 Department of Surgery, Uppsala University Hospital, Uppsala, Sweden

## Abstract

**Background:**

The success of modern multimodal treatment in rectal cancer is dependent on risk prediction. Better knowledge of the risk of locoregional and distant recurrence, in relation to preoperative treatment, pathological stage, and commonly used risk factors, is needed when deciding on adjuvant therapy and surveillance.

**Methods:**

The Swedish ColoRectal Cancer Registry was used to identify patients diagnosed with rectal adenocarcinoma between 2011 and 2018. Readily available variables, including patient, tumor, and treatment factors were exposures. Cox proportional hazard models were used to identify important risk factors for recurrence and calculate recurrence risks.

**Results:**

A total of 9428 curatively resected patients were included and followed for a median of 72 months. Eighteen percent had distal recurrence and 3% had locoregional recurrence at 5 years. Risk factors with major impact on distal recurrence were pT4a (hazard ratio [HR] 5.1, 95% confidence interval [CI] 3.3–8.0), pN2b (HR 3.4, 95% CI 2.7–4.2), tumor deposit (HR 1.7, 95% CI 1.5–1.9), lymph node yield (HR 1.5, 95% CI 1.3–1.8), and tumor level 0–5 cm (HR 1.5, 95% CI 1.3–1.8). Pathologic stage and number of risk factors identified groups with markedly different recurrence risks in all neoadjuvant treatment groups.

**Conclusions:**

Readily available risk factors, as a complement to stage, are still valid and robust in all neoadjuvant treatment groups. Tumor deposit is important, while circumferential resection margin might no longer be important with improved oncological treatments and high-quality TME surgery. Tailored surveillance is possible in selected groups using risk stratification based on stage and risk factors.

**Supplementary Information:**

The online version contains supplementary material available at 10.1245/s10434-024-15552-x.

Over recent decades, significant advancements have been made in the treatment of rectal cancer. Better staging through the means of magnetic resonance imaging (MRI), individualized neoadjuvant oncological treatment, and specimen-oriented surgery have contributed to reduced recurrence rates and lengthened survival.^1,2^ While locoregional recurrence (LRR) now only concerns a minority of patients, systemic recurrences persist as the leading cause of mortality.^2,3^ Consequently, endeavors have been focused on implementing systemic therapies to reduce distant metastases (DM) and thereby enhance survival. The success of these treatments is highly dependent on risk prediction, i.e., to give oncological treatment to patients with sufficient risk of recurrence. Pretreatment MRI, as demonstrated by the findings of e.g., the MERCURY trial, has proven effective in predicting the risk of LRR and survival.^4^ Yet, the accuracy of risk prediction by clinical, pretherapeutic characteristics are still inferior to that of posttherapy pathologic stage, especially after neoadjuvant treatments.^5^ Novel treatment regimens, such as total neoadjuvant therapy (TNT), appear to induce further downstaging which adds additional complexities in the prognostication.^1,6,7^

While both NCCN (National Comprehensive Cancer Network) and ESMO (European Society for Medical Oncology) advocate risk prediction based on well-established histopathological features in colon cancer, these recommendations are uncertain for rectal cancer.^8–11^ Studies have mostly either been conducted in a single institution setting or focused on one risk factor (RF). Models for the prediction of recurrence have been constructed, but these were done during an era when the use of conventional chemoradiotherapy (CRT) for 5 weeks and adjuvant therapy was more prevalent, and the importance of factors, such as vascular and perineural invasion and tumor deposits (TD), were less known.^12^ Better knowledge of (pathological) stage-specific risk for recurrence and death, stratified by type of neoadjuvant treatment and RFs, is needed and may aid clinicians in their decision-making regarding the need for adjuvant treatment and surveillance.

Our goals were to identify the most important risk factors for DM and LRR and assess the risks in pathological stages 0 to III in a large population-based national cohort treated according to up-to-date principles after MRI-staging and where adherence to guidelines is high. Additionally, we explored whether the same risk factors for recurrence apply universally, irrespective of neoadjuvant treatment, or if the treatment alters the risk profile.

## Methods

The Strengthening the reporting of observational studies in epidemiology (STROBE) checklist for the reporting of observational studies was followed.^13^

### Patients and Study Design

**Fig. 1:**
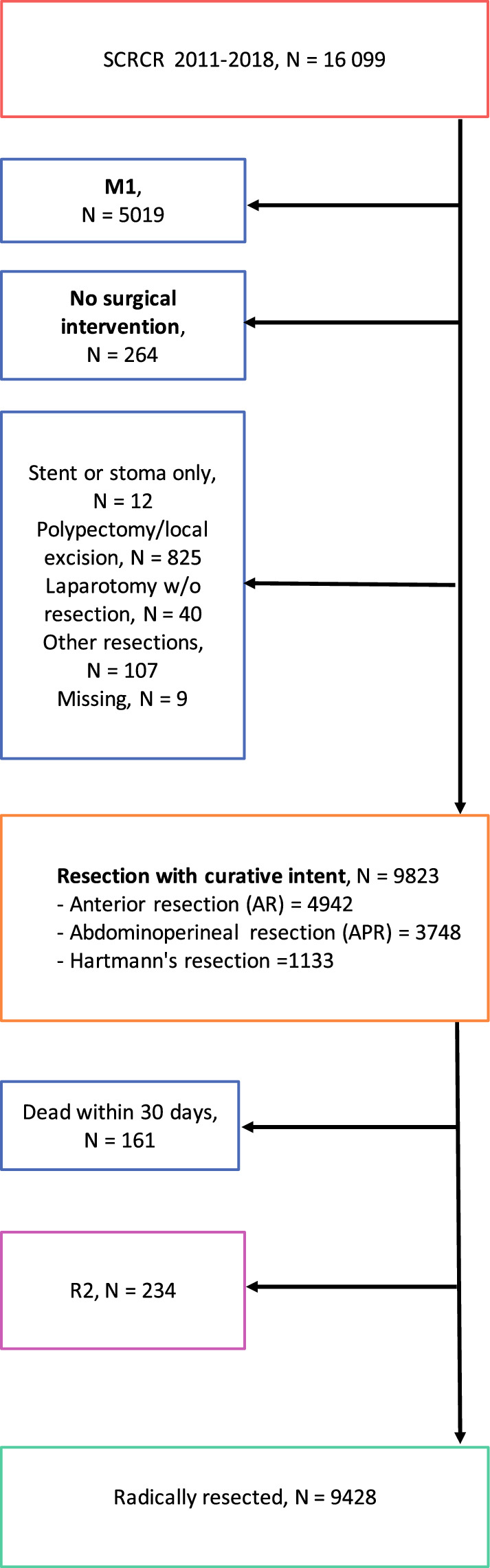
Flow chart of the cohort and selection of patients from the Swedish ColoRectal Cancer Registry (SCRCR). The study included all patients diagnosed between 2011 and 2018. *M*1 distant metastasis; *R*2 macroscopically nonradical surgery defined by the surgeon

The Swedish ColoRectal Cancer Registry (SCRCR) is a prospectively maintained nationwide quality registry for all CRC cases.^14^ The registry has been externally and internally validated.^15–17^ For this study, data were retrieved from SCRCR for all cases of rectal cancer between 2011 and 2018 (*n* = 16,099) to achieve complete registration of relevant variables and at least 3 years of follow-up. Exclusion was done per the flow chart (Fig. 1), leaving those with stage 0–III disease who underwent curative resection and survived at least 30 days after surgery (*n* = 9428). Outcome variables, including LRR, DM, and mortality were presented in terms of incidence (%). Time to LRR, time to DM, disease-free (DFS), and overall survival (OS) were calculated from the time of surgery until the occurrence of respective events. Disease-free survival and OS were censored at the end of the follow-up if still alive.

Risk factors for recurrence stated in the NCCN and ESMO guidelines for colon cancer and other known RFs for rectal cancer were established as exposure variables.^8,9^ These consisted of (y)pT and (y)pN classifications, lymph node yield (< 12), circumferential resection margin (CRM), presence of TD, differentiation, vascular and perineural invasion, mucinous feature, tumor level (distance from anal verge measured during rigid rectoscopy) and presence of perforation. pT3 (tumors invading the submucosa) was subcategorized into pT3ab (< 5-mm invasion) and pT3cd (≥ 5-mm invasion). Additional exposure variables covering patient-related data (age, sex, and comorbidities) and treatment-related variables, such as type of oncological and surgical treatment were included. Tumor staging followed the TNM classification system, 7th edition.^18^

Stratification of neoadjuvant and adjuvant treatment was done based on contemporary treatment regimens. Whenever feasible, additional stratification was implemented to facilitate more detailed analysis, exemplified by the distinction between short-course radiotherapy with surgery within a week (scRT) and surgery delayed by 4–8+ weeks (scRT-delay). Conventional chemoradiotherapy and TNT, having similarities in patient selection and treatment approaches, were combined for certain calculations because of infrequent use of TNT, limiting power in subanalysis. A less-detailed pT-categorization was needed when stratifying by pretreatment because of a low number of events per group. Adjuvant chemotherapy was grouped into fluoropyrimidine (FU) with or without oxaliplatin, irrespective of the means of FU administration.

### Statistics

Group distributions were described and differences in recurrence and survival were tested with the two-sided Pearson χ^2^ test. Survival analysis was conducted by using Cox proportional hazard models and were reported as hazard ratios (HRs) for recurrence, OS, and disease-free survival (DFS). Clinically relevant variables were included in the multivariable models. No imputation was performed and adjusted analyses were done by using complete case analysis.

Noncollinearity was tested using variance influential factor, revealing collinearity between tumor level and surgical treatment. Tumor level was selected for inclusion in the multivariable regression, recognizing that the choice of surgical procedure may be influenced by various regional and cultural factors. The proportionality hazard assumption was controlled through visual inspection of the Schoenfeld residuals. The reverse Kaplan-Meier estimator was used for calculating median follow-up time.^19^

Recurrences at 5 years, stratified by the number of RFs derived from the Cox proportional hazard models, were calculated for each stage. Further stratification was done for recurrence in (y)pT and (y)pN subgroups. Kaplan-Meier curves were plotted for stages 0–III stratified by the number of RFs. Differences between groups were tested for significance through the Mantel-Cox log-rank test.

All statistical calculations were performed by using R (version 4.3.1, R Core Team 2023), R Studio^®^ (Rstudio Team, 2023). Differences were considered statistically significant for *p* values ≤ 0.05.

## Results

The median age of the cohort was 70 years (range 14–96). Median follow-up was 75 months (interquartile range [IQR] 53–99). Demography, pathology, and treatment data for the cohort are summarized in Table 1, along with the distribution of LRR, DM, and mortality. The recurrence rate at 5 years was 19% for the entire cohort, with stage-specific rates of 9% in stage 0, 8% in stage I, 18% in stage II, and 34% in stage III. The 5-year rate of DM was 18%, with stage-specific rates of 7%, 7%, 16%, and 32% for stages 0 to III, respectively. The 5-year rate of LRR was 3% in total and 2%, 2%, 3%, and 6% for stages 0 to III, respectively. Of the 287 patients with LRR, 72 (25%) had DM before or within 60 days following the LRR diagnosis.

**Table 1:** Distribution of characteristics, number of recurrences, and mortality in the cohort

Characteristic	Overall, *N* = 9428	LRR, *N* = 287		DM, *N* = 1561		Mortality, *N* = 2537		
	*N* (% column)	*N* (% row)	*p*^1^	*N* (% row)	*p*^1^	*N* (% row)		*p*^1^
Sex			0.9		0.11			**< 0.001**
Female	3761 (40%)	113 (3%)		594 (16%)		922 (25%)		
Male	5667 (60%)	174 (3%)		966 (17%)		1614 (28%)		
Age, years			0.4		**0.008**			**< 0.001**
< 75	6638 (70%)	196 (3%)		1134 (17%)		1293 (19%)		
≥ 75	2789 (30%)	91 (3%)		425 (15%)		1242 (45%)		
ASA			0.3		>0.9			**< 0.001**
1	1725 (18%)	45 (3%)		291 (17%)		258 (15%)		
2	5311 (56%)	165 (3%)		878 (17%)		1267 (24%)		
3	2168 (23%)	68 (3%)		358 (17%)		919 (42%)		
4	106 (1%)	2 (2%)		16 (15%)		64 (60%)		
Unknown	118 (1%)	7 (6%)		17 (14%)		28 (24%)		
Surgery			**< 0.001**		**< 0.001**			**< 0.001**
AR	4813 (51%)	110 (2%)		700 (15%)		940 (20%)		
APR	3559 (38%)	135 (4%)		700 (20%)		1104 (31%)		
Hartmann	1056 (11%)	42 (4%)		160 (15%)		492 (47%)		
Tumor level (cm)			**< 0.001**		**< 0.001**			**< 0.001**
10–15	2743 (29%)	61 (2%)		389 (14%)		692 (25%)		
5–10	3926 (42%)	119 (3%)		627 (16%)		999 (25%)		
0–5	2657 (28%)	99 (4%)		520 (20%)		806 (30%)		
Unknown	102 (1%)	8 (8%)		24 (24%)		39 (38%)		
AJCC stage			**< 0.001**		**< 0.001**			**< 0.001**
I	3012 (32%)	45 (1%)		198 (7%)		529 (18%)		
0	282 (3%)	4 (1%)		21 (7%)		41 (15%)		
II	3076 (33%)	91 (3%)		459 (15%)		818 (27%)		
III	3022 (32%)	146 (5%)		877 (29%)		1140 (38%)		
Unknown	36 (0%)	1 (3%)		5 (14%)		8 (22%)		
(y)pT			**< 0.001**		**< 0.001**			**< 0.001**
T1	831 (9%)	8 (1%)		42 (5%)		136 (16%)		
T0	328 (3%)	5 (2%)		31 (9%)		54 (16%)		
T2	2879 (31%)	45 (2%)		272 (9%)		574 (20%)		
T3ab	2879 (31%)	85 (3%)		506 (18%)		800 (28%)		
T3cd	1296 (14%)	63 (5%)		366 (28%)		475 (37%)		
T3 unknown	616 (7%)	35 (6%)		161 (26%)		239 (39%)		
T4a	262 (3%)	21 (8%)		83 (32%)		115 (44%)		
T4b	248 (3%)	16 (6%)		71 (29%)		105 (42%)		
T4 unknown	49 (1%)	8 (16%)		22 (45%)		28 (57%)		
Unknown	40 (0%)	1 (3%)		6 (15%)		10 (25%)		
(y)pN			**< 0.001**		**< 0.001**			**< 0.001**
N0	6281 (67%)	133 (2%)		665 (11%)		1365 (22%)		
N1a	1065 (11%)	44 (4%)		236 (22%)		324 (30%)		
N1b	961 (10%)	39 (4%)		262 (27%)		337 (35%)		
N2a	556 (6%)	25 (4%)		177 (32%)		241 (43%)		
N2b	440 (5%)	38 (9%)		202 (46%)		238 (54%)		
Unknown	125 (1%)	8 (6%)		18 (14%)		31 (25%)		
Lymph node yield			**< 0.001**		**0.004**			**< 0.001**
≥ 12	8037 (85%)	228 (3%)		1288 (16%)		2064 (26%)		
< 12	1302 (14%)	50 (4%)		257 (20%)		445 (34%)		
Unknown	89 (1%)	9 (10%)		15 (17%)		27 (30%)		
Tumor deposit			**< 0.001**		**< 0.001**			**< 0.001**
No	7519 (80%)	176 (2%)		1000 (13%)		1759 (23%)		
Yes	1145 (12%)	77 (7%)		419 (37%)		476 (42%)		
Unknown	764 (8%)	34 (4%)		141 (18%)		301 (39%)		
Perforation			**< 0.001**		**0.004**			**0.008**
No perforation	8976 (95%)	259 (3%)		1475 (16%)		2384 (27%)		
Perf not near tumor	195 (2%)	9 (5%)		26 (13%)		63 (32%)		
Perf near tumor	171 (2%)	16 (9%)		45 (26%)		62 (36%)		
Unknown	86 (1%)	3 (3%)		14 (16%)		27 (31%)		
Differentiation			**< 0.001**		**< 0.001**			**< 0.001**
High/moderate	7339 (78%)	185 (3%)		1135 (15%)		1893 (26%)		
Poor	1194 (13%)	64 (5%)		269 (23%)		388 (32%)		
Unknown	895 (9%)	38 (4%)		156 (17%)		255 (28%)		
CRM (mm)			**< 0.001**		**< 0.001**			**< 0.001**
≥ 2	7891 (84%)	189 (2%)		1229 (16%)		2029 (26%)		
1.1–1.9	836 (9%)	25 (3%)		122 (15%)		217 (26%)		
≤ 1	659 (7%)	72 (11%)		206 (31%)		283 (43%)		
Unknown	42 (0%)	1 (2%)		3 (7%)		7 (17%)		
Mucinous			**< 0.001**		**< 0.001**			**< 0.001**
No	8155 (86%)	228 (3%)		1282 (16%)		2108 (26%)		
Yes	925 (10%)	47 (5%)		207 (22%)		316 (34%)		
Unknown	348 (4%)	12 (3%)		71 (20%)		112 (32%)		
Vascular invasion			**<** **0.001**		**< 0.001**			**< 0.001**
No	7018 (74%)	158 (2%)		928 (13%)		1696 (24%)		
Yes	2189 (23%)	119 (5%)		603 (28%)		780 (36%)		
Unknown	221 (2%)	10 (5%)		29 (13%)		60 (27%)		
Perineural invasion			**< 0.001**		**< 0.001**			**< 0.001**
No	7513 (80%)	178 (2%)		987 (13%)		1777 (24%)		
Yes	1545 (16%)	95 (6%)		522 (34%)		639 (41%)		
Unknown	370 (4%)	14 (4%)		51 (14%)		120 (32%)		
Neoadjuvant treatment			**< 0.001**		**< 0.001**			**< 0.001**
Surgery only	3415 (36%)	106 (3%)		423 (12%)		916 (27%)		
scRT	2786 (30%)	56 (2%)		463 (17%)		723 (26%)		
scRT-delay	1190 (13%)	42 (4%)		231 (19%)		412 (35%)		
CRT	1340 (14%)	50 (4%)		286 (21%)		326 (24%)		
TNT	334 (4%)	14 (4%)		69 (21%)		56 (17%)		
Unknown	363 (4%)	19 (5%)		88 (24%)		103 (28%)		
Adjuvant chemotherapy			**< 0.001**		**< 0.001**			**0.009**
None	7228 (77%)	196 (3%)		989 (14%)		1999 (28%)		
Only FU	1172 (12%)	42 (4%)		284 (24%)		280 (24%)		
FU + Oxaliplatin	1028 (11%)	49 (5%)		287 (28%)		257 (25%)		

Bold values denote statistical significance at the *p* < 0.05

*LRR* locoregional recurrence; *DM* distant metastatis; *ASA* American Society of Anethesiologists Physical Status; *AJCC* American Joint Committee on Cancer; *AR* anterior resection; *APR* abdominoperineal resection; *Perf* perforation; *CRM* circumferential resection margin; *scRT* short-course radiotherapy; *scRT-delay* scRT with delay to surgery; *CRT* chemoradiotherapy; *TNT* total neoadjuvant therapy; *FU* fluoropyrimidine

^1^Pearson’s chi-squared test

Stage 0 after preoperative treatment was 0% after surgery and scRT, 7% after scRT-delay, 9% after CRT, and 15% after TNT. The rates of both DM and LRR differed significantly between AJCC stages, (y)pT-stages, and (y)pN-stages (Table 1). The rates were identical between stages 0 and I.

The 5-year OS was 90% for stage 0, 86% for stage I, 78% for stage II, and 67% for stage III. Disease-free survival rates demonstrated a similar stage-wise distribution with 85%, 82%, 70%, and 56% for stages 0 to III, respectively.

### Risk Factors of Recurrence

Uni- and multivariable Cox proportional hazards for recurrences are presented in Table 2 (OS and DFS in Table S1). All factors, except age and ASA, correlated with an increased risk of DM in the unadjusted regression. The same applied to LRR, with the added exception of sex. Besides stage variables, TD and perineural invasion had the highest HRs for DM, and CRM ≤ 1 mm, perforation near the tumor, and TD had the highest HRs for LRR.

**Table 2:** Cox proportional hazards for recurrence

Characteristic	Univariable								Multivariable							
	LRR				DM				LRR				DM			
	*N* events	HR	95% CI	*p*^1^	*N* events	HR	95% CI	*p*^1^	*N* events	HR	95% CI	*p*^1^	*N* events	HR	95% CI	*p*^1^
Sex	287			0.72	1559			**0.046**	200			0.38	1155			0.20
Female		Ref	Ref			Ref	Ref			Ref	Ref			Ref	Ref	
Male		1.04	0.82, 1.32			1.11	1.00, 1.23			1.14	0.85, 1.52			1.08	0.96, 1.22	
Age, years				0.14				0.25				0.36				0.18
< 75		Ref	Ref			Ref	Ref			Ref	Ref			Ref	Ref	
≥ 75		1.21	0.94, 1.55			0.94	0.84, 1.05			1.18	0.83, 1.66			0.91	0.78, 1.05	
ASA				0.35				0.51				0.66				0.91
1		Ref	Ref			Ref	Ref			Ref	Ref			Ref	Ref	
2		1.26	0.90, 1.75			1.02	0.89, 1.16			1.01	0.68, 1.50			0.98	0.84, 1.15	
3		1.39	0.96, 2.03			1.10	0.95, 1.29			1.07	0.68, 1.70			0.98	0.81, 1.18	
4		0.98	0.24, 4.06			1.16	0.70, 1.92			0.37	0.05, 2.78			1.19	0.70, 2.02	
Surgery				**< 0.001**				**< 0.001**								
AR		Ref	Ref			Ref	Ref									
APR		1.77	1.37, 2.27			1.45	1.30, 1.61									
Hartmann		2.02	1.42, 2.89			1.17	0.98, 1.38									
Tumor level (cm)				**0.003**				**< 0.001**				**< 0.001**				**< 0.001**
11–15		Ref	Ref			Ref	Ref			Ref	Ref			Ref	Ref	
6–10		1.39	1.02, 1.89			1.15	1.02, 1.31			1.68	1.14, 2.45			1.20	1.03, 1.40	
0–5		1.73	1.26, 2.38			1.44	1.27, 1.65			2.32	1.54, 3.49			1.48	1.25, 1.75	
AJCC stage				**< 0.001**				**< 0.001**								
I		Ref	Ref			Ref	Ref									
0		0.93	0.34, 2.60			1.13	0.72, 1.77									
II		2.06	1.44, 2.95			2.45	2.07, 2.89									
III		3.57	2.56, 4.99			5.29	4.53, 6.17									
pT				**< 0.001**				**< 0.001**				**< 0.001**				**< 0.001**
T1		Ref	Ref			Ref	Ref			Ref	Ref			Ref	Ref	
T0		1.57	0.51, 4.79			1.90	1.20, 3.02			1.73	0.19, 15.5			2.11	0.91, 4.92	
T2		1.63	0.77, 3.46			1.92	1.38, 2.65			1.75	0.68, 4.53			1.84	1.25, 2.70	
T3ab		3.20	1.55, 6.61			3.80	2.78, 5.21			3.42	1.36, 8.62			3.02	2.07, 4.41	
T3cd		5.61	2.69, 11.7			6.81	4.95, 9.38			4.25	1.63, 11.0			3.90	2.64, 5.77	
T3 unknown		6.03	2.80, 13.0			5.77	4.11, 8.10			4.81	1.76, 13.1			3.80	2.49, 5.80	
T4a		9.94	4.40, 22.4			8.06	5.56, 11.7			6.48	2.22, 18.9			5.12	3.27, 8.03	
T4b		8.25	3.53, 19.3			7.50	5.12, 11.0			3.33	1.08, 10.3			3.88	2.43, 6.20	
T4 unknown		22.4	8.41, 59.7			13.9	8.32, 23.3			16.0	4.63, 55.5			10.8	5.87, 19.7	
pN				**< 0.001**				**< 0.001**				0.076				**< 0.001**
N0		Ref	Ref			Ref	Ref			Ref	Ref			Ref	Ref	
N1a		2.03	1.45, 2.86			2.27	1.96, 2.64			1.50	1.00, 2.25			1.83	1.53, 2.18	
N1b		2.07	1.45, 2.96			2.98	2.58, 3.43			1.09	0.67, 1.77			2.14	1.79, 2.56	
N2a		2.32	1.51, 3.56			3.55	3.00, 4.18			1.58	0.94, 2.65			2.29	1.85, 2.84	
N2b		4.89	3.41, 7.01			5.79	4.95, 6.79			1.93	1.14, 3.25			3.38	2.72, 4.20	
Lymph node yield				**0.041**				**< 0.001**				0.11				**< 0.001**
≥ 12		Ref	Ref			Ref	Ref			Ref	Ref			Ref	Ref	
< 12		1.39	1.03, 1.89			1.28	1.12, 1.47			1.39	0.94, 2.06			1.53	1.30, 1.81	
Tumor deposit				**< 0.001**				**< 0.001**				**< 0.001**				**< 0.001**
No		Ref	Ref			Ref	Ref			Ref	Ref			Ref	Ref	
Yes		3.23	2.47, 4.22			3.42	3.05, 3.83			1.78	1.27, 2.50			1.67	1.45, 1.93	
Perforation				**< 0.001**				**< 0.001**				**0.010**				0.056
No perforation		Ref	Ref			Ref	Ref			Ref	Ref			Ref	Ref	
Perf not near tumor		1.70	0.87, 3.31			0.83	0.56, 1.23			1.36	0.63, 2.94			0.61	0.38, 0.99	
Perf near tumor		3.66	2.21, 6.06			1.81	1.34, 2.43			2.70	1.51, 4.82			1.21	0.85, 1.72	
Differentiation				**< 0.001**				**< 0.001**				0.10				0.57
High/moderate		Ref	Ref			Ref	Ref			Ref	Ref			Ref	Ref	
Poor		2.28	1.71, 3.03			1.58	1.38, 1.80			1.36	0.95, 1.94			0.95	0.81, 1.12	
CRM (mm)				**< 0.001**				**< 0.001**				**< 0.001**				0.74
≥ 2		Ref	Ref			Ref	Ref			Ref	Ref			Ref	Ref	
1.1–1.9		1.24	0.82, 1.88			0.92	0.77, 1.11			1.36	0.76, 2.46			1.01	0.78, 1.31	
≤ 1		5.39	4.11, 7.07			2.42	2.09, 2.80			2.50	1.73, 3.62			1.08	0.89, 1.31	
Mucinous				**< 0.001**				**< 0.001**				0.10				0.51
No		Ref	Ref			Ref	Ref			Ref	Ref			Ref	Ref	
Yes		1.91	1.39, 2.61			1.52	1.31, 1.76			1.40	0.94, 2.07			1.06	0.88, 1.28	
Perineural invasion				**< 0.001**				**< 0.001**				0.55				**< 0.001**
No		Ref	Ref			Ref	Ref			Ref	Ref			Ref	Ref	
Yes		2.95	2.30, 3.79			3.13	2.81, 3.48			1.11	0.78, 1.58			1.40	1.22, 1.62	
Vascular invasion				**< 0.001**				**<0.001**				**0.018**				**< 0.001**
No		Ref	Ref			Ref	Ref			Ref	Ref			Ref	Ref	
Yes		2.68	2.11, 3.40			2.42	2.19, 2.68			1.49	1.07, 2.08			1.28	1.12, 1.47	
Neoadjuvant treatment				**< 0.001**				**< 0.001**				**< 0.001**				**< 0.001**
Surgery only		Ref	Ref			Ref	Ref			Ref	Ref			Ref	Ref	
scRT		0.63	0.46, 0.87			1.33	1.17, 1.52			0.42	0.28, 0.62			0.93	0.80, 1.10	
scRT-delay		1.22	0.85, 1.74			1.71	1.46, 2.01			1.04	0.68, 1.58			1.44	1.19, 1.73	
CRT		1.30	0.95, 1.78			1.83	1.58, 2.11			0.78	0.50, 1.20			1.29	1.07, 1.56	
TNT		1.46	0.84, 2.56			1.88	1.46, 2.42			0.93	0.45, 1.93			1.58	1.16, 2.14	
Adjuvant treatment				**0.005**				**< 0.001**				0.45				**0.006**
None		Ref	Ref			Ref	Ref			Ref	Ref			Ref	Ref	
Only FU		1.28	0.92, 1.78			1.81	1.59, 2.06			0.76	0.49, 1.17			0.94	0.80, 1.11	
FU + oxaliplatin		1.69	1.24, 2.31			2.10	1.84, 2.39			0.92	0.58, 1.46			0.73	0.61, 0.89	

Bold values denote statistical significance at the *p* < 0.05

*HR* Hazard ratio; *CI* confidence interval^*;*^* LRR* locoregional recurrence; *DM* distant metastatis; *ASA* American Society of Anethesiologists Physical Status; *AJCC* American Joint Committee on Cancer; *AR* anterior resection. *APR* abdominoperineal resection; *Perf* perforation; *CRM* circumferential resection margin; *scRT* short-course radiotherapy; scRT-delay scRT with delay to surgery; *CRT* chemoradiotherapy; *TNT* total neoadjuvant therapy; FU fluoropyrimidine

^1^Pearson’s chi-squared test

In the adjusted analyses, factors correlating with DM were (y)pT, (y)pN, lymph node yield, tumor level, TD, perineural invasion, vascular invasion, neoadjuvant treatment, and adjuvant treatment. The highest risks were seen for (y)pT4a, (y)pN2b, tumor deposit, lymph node yield, and tumor level 0–5 cm (Table 2). Factors correlating with LRR were tumor level, (y)pT, TD, perforation, CRM, vascular invasion, and neoadjuvant treatment (Table 2).

### Stratification by Neoadjuvant Treatment

Regressions for DM and LRR stratified by pretreatment are presented in Supplementary Table S2a and b. Factors associated with DM, regardless of neoadjuvant treatment, were the American Joint Committee on Cancer (AJCC) stage, (y)pT, (y)pN, TD, differentiation, CRM, perineural invasion, vascular invasion, and adjuvant treatment (Table S2a). A separate regression analysis was done for patients who received CRT (no adjuvant treatment is provided after TNT), confirming the correlation with adjuvant treatment. Lymph node yield was statistically significant only in the two groups without delay to surgery (i.e., surgery alone and scRT) and perforation only in patients operated directly.

For LRR, unadjusted factors with associations regardless of group affiliation were AJCC stage, (y)pT, TD, CRM, and perineural invasion (Table S2b).

Only (y)pT and (y)pN were statistically significant in all treatment groups in the multivariable regressions for DM, whereas sex, age, ASA, differentiation, CRM, and vascular invasion were not significant in any of the groups (Table S2a; Fig. S1). In concordance with the univariable regressions, lymph node yield was only statistically significant in the two groups without delay to surgery. Tumor deposits and perineural invasion were associated with an increased risk of DM in all but the scRT-delay group.

No risk factor was statistically significant in all groups in the adjusted regression for LRR (Table S2b).

The 5-year DM rates (%) in the surgery only, scRT, scRT-delay, CRT, and TNT groups were 14, 18, 23, 23, and 24, respectively. Locoregional recurrence rates (%) for the groups, in the same order, were 4, 2, 4, 4, and 5.

Generally, patients in the surgery only group had less advanced tumors, exemplified by lower AJCC stage. The tumors had higher differentiation, a larger CRM was achieved, and the rate of DM was lower compared with the groups that received neoadjuvant treatment. Patients in the scRT-delay group were older, had more comorbidities (higher ASA), were more often operated with Hartmann’s surgery, and received adjuvant chemotherapy to a lesser extent compared to the other groups. Patients in the two groups without delay to surgery had more often a lymph node yield ≥ 12 (*p* < 0.001). Characteristics of the treatment groups can be found in Supplementary Table S4.

### Rate of DM and Locoregional Recurrence According to Stage and Number of Risk Factors

Independent RFs derived from the multivariable Cox model were used to compare 5-year DM rates stratified by the number of RFs and grouped by oncological treatment (Tables 3 and S3a for further subdivisions).

**Table 3:** DM rates at 5 years after surgery stratified by stage and RF

	RF	Overall		Surgery only		scRT		scRT-delay		CRT/TNT	
		*n*	% (95% CI, %)	*n*	% (95% CI, %)	*n*	% (95% CI, %)	*n*	% (95% CI, %)	*n*	% (95% CI, %)
Stage											
0	0	120	4 (1, 8)			3	33 (0, 70)	34	3 (0, 8)	81	4 (0, 8)
	1	125	9 (4, 14)			5	20 (0, 48)	40	5 (0, 12)	78	9 (2, 15)
	≥ 2	37	11 (0, 20)			3	0 (0, 0)	9	11 (0, 29)	25	12 (0, 24)
I	0	1544	6 (4, 7)	921	4 (2, 5)	351	7 (4, 10)	130	11 (4, 17)	134	11 (5, 16)
	1	1077	8 (6, 9)	383	7 (4, 9)	352	8 (5, 11)	157	8 (3, 12)	174	9 (5, 14)
	≥ 2	391	12 (9, 16)	104	17 (9, 25)	150	9 (4, 14)	57	13 (2, 23)	74	11 (4, 19)
II	0	1293	11 (9, 13)	536	11 (8, 14)	393	8 (5, 10)	139	18 (11, 25)	199	14 (9, 19)
	1	998	14 (11, 16)	281	12 (7, 16)	312	10 (6, 13)	142	22 (14, 30)	243	18 (12, 22)
	≥ 2	785	28 (24, 31)	173	30 (22, 37)	243	25 (19, 30)	126	37 (27, 46)	221	26 (19, 32)
III	0	597	15 (12, 18)	260	14 (9, 19)	170	12 (7, 17)	77	18 (8, 26)	86	19 (10, 27)
	1	876	24 (21, 27)	312	18 (13, 22)	311	23 (18, 28)	79	33 (20, 44)	161	32 (24, 39)
	≥ 2	1548	43 (40, 46)	434	38 (32, 43)	568	39 (35, 43)	198	50 (41, 58)	323	53 (46, 58)

	RF	Overall		No adjuvant Tx		Adjuvant Tx		Surgery only and no adjuvant Tx		Surgery only and adjuvant Tx	
		*n*	% (95% CI, %)	*n*	% (95% CI, %)	*n*	% (95% CI, %)	*n*	% (95% CI, %)	*n*	% (95% CI, %)
Stage											
0	0	120	4 (1, 8)	100	4 (0, 8)	20	5 (0, 14)				
	1	125	9 (4, 14)	115	8 (3, 13)	10	21 (0, 44)				
	≥ 2	37	11 (0, 20)	28	4 (0, 10)	9	33 (0, 58)				
I	0	1544	6 (4, 7)	1506	5 (4, 7)	38	13 (2, 24)	913	3 (2, 5)	8	14 (0, 37)
	1	1077	8 (6, 9)	1016	7 (6, 9)	61	14 (4, 22)	366	6 (3, 9)	17	19 (0, 36)
	≥ 2	391	12 (9, 16)	344	12 (8, 16)	47	14 (3, 24)	89	18 (8, 26)	15	14 (0, 30)
II	0	1293	11 (9, 13)	1227	11 (9, 12)	66	22 (11, 31)	521	10 (7, 13)	15	40 (9, 60)
	1	998	14 (11, 16)	846	13 (10, 15)	152	18 (11, 24)	242	12 (8, 17)	39	8 (0, 17)
	≥ 2	785	28 (24, 31)	557	27 (23, 31)	228	30 (23, 36)	127	25 (16, 33)	46	42 (25, 56)
III	0	597	15 (12, 18)	325	18 (13, 22)	272	12 (8, 15)	131	20 (12, 28)	129	9 (4, 14)
	1	876	24 (21, 27)	425	27 (23, 32)	451	21 (17, 25)	154	27 (18, 34)	158	10 (5, 15)
	≥ 2	1548	43 (40, 46)	704	47 (43, 51)	844	40 (36, 43)	212	44 (35, 51)	222	33 (26, 40)

Risk factors (RF) for DM: (y)pT4, (y)pN2, perineural invasion, vascular invasion, tumor deposit, lymph node yield, and tumor level 0–5 cm

*scRT* Short-course radiotherapy; *scRT-delay* scRT with delay to surgery; *CRT* chemoradiotherapy; *TNT* total neoadjuvat therapy; Stage according to American Joint Committee on Cancer (AJCC). *DM* distant metastatis; *Ref* reference; *Tx* treatment; *CI* confidence interval

Distant recurrence rates were significantly higher with each stage and doubled between patients with 0 and ≥ 2 RFs. Statistically significant differences were found between 1 and ≥ 2 RFs for stages I–III (*p* < 0.001) but not for stage 0. However, in stage 0, numerically higher DM rates were seen when RFs were present.

**Fig. 2:**
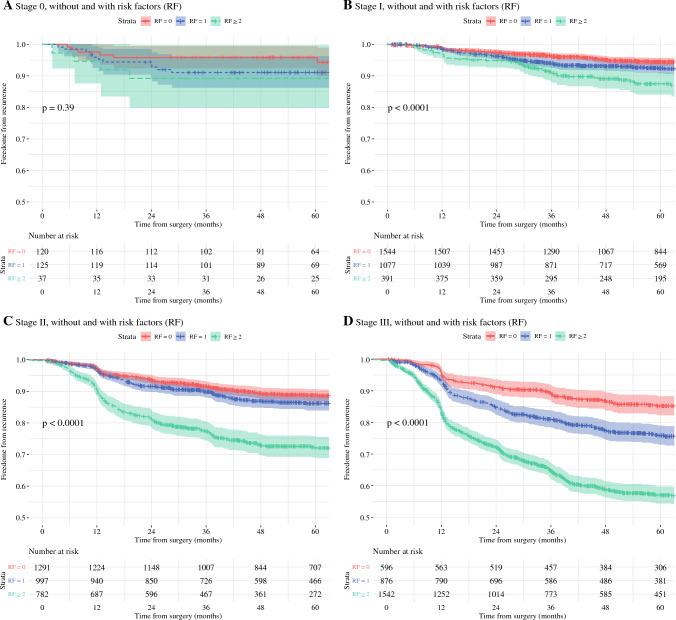
Kaplan-Meier estimates for DM stratified by AJCC stages and RFs. **A** Stage 0; **B** Stage I; **C** Stage II; **D** Stage III. *DM* distant metastasis; *AJCC* American Joint Committee on Cancer; *RF* risk factors

Patients in stage II with ≥ 2 RFs had a significantly higher DM rate compared with patients in stage III with 0 RF (*p* < 0.001). Rates within each stage are visualised in Kaplan–Meier’s estimates (Fig. 2).

Independent RFs were used to compare 5-year LRR rates stratified by number of RFs and grouped by oncological treatment (Table S3b). The RFs comprised of (y)pT4, TD, differentiation, mucinous feature, and tumor levels 0–5 cm and 6–10 cm. Overall, 5-year LRR rates remained below 5% across all stages and RFs except for stage III with two or more RFs.

**Fig. 3:**
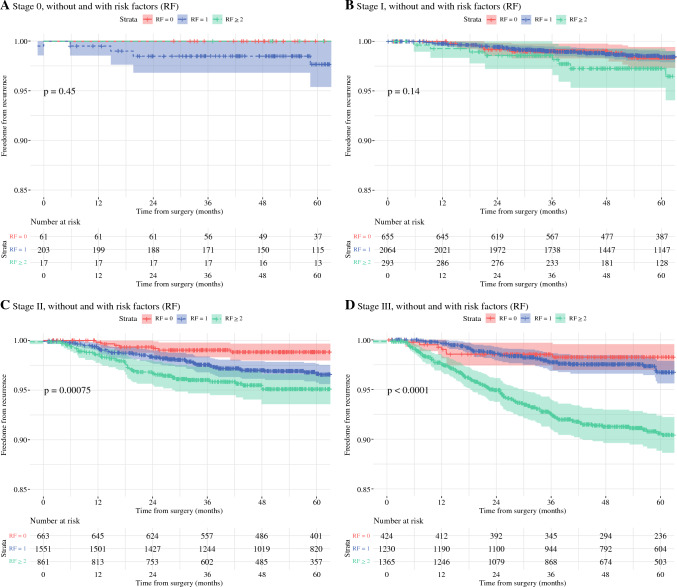
Kaplan-Meier estimates for LRR stratified by AJCC stages and RFs. **A** Stage 0. **B** Stage I; **C** Stage II; **D** Stage III. *LRR* locoregional recurrence; *AJCC* American Joint Committee on Cancer; *RFs* risk factors

Statistically significant differences were found between 0 and 1 RF for stages II and III (*p* < 0.05) and between 1 and ≥ 2 RFs for stages I–III (*p* < 0.001). Rates more than doubled between patients with 0 and ≥ 2 RFs in stages II-III. LRR rates within each stage are visualised in Kaplan–Meier’s estimates (Fig. 3).

## Discussion

In this comprehensive evaluation of all routinely recorded factors in a whole-population setting, we found that while clinical variables were of some importance, pathological stage, along with other pathological characteristics and tumor level contributed most to predicting DM.

Independent RFs for DM, ordered by importance, included (y)pT, (y)pN, TD, lymph node yield, tumor level, vascular invasion, and perineural invasion. These factors demonstrated consistent strength across most groups defined by the preoperative treatment, emphasizing robust clinical relevance. The fact that the DM rate within each stage doubled between patients with 0 and ≥ 2 RFs is noteworthy. Except for vascular invasion, these RFs also displayed statistical significance for both DFS and OS when adjusting for all included variables.

For LRR, the most important RFs were (y)pT, CRM, tumor level, TD, perforation, and vascular invasion. However, the statistical significance of these factors varied when analyzed on a group basis, probably because of a low number of events.

Unsurprisingly, the highest DM rates were found in patients with the combination of advanced AJCC stages and two or more RFs, particularly after neoadjuvant therapy. The identification of these less-therapy-sensitive tumors is important and should be carefully evaluated for additional therapies and closer monitoring. When adjusting for all included variables, HR for adjuvant chemotherapy was 0.9 for fluorouracil alone and 0.7 with the addition of oxaliplatin. Unlike for colon cancer, the efficacy of adjuvant chemotherapy in rectal cancer is controversial and the risk reduction indicated in the present study must be interpreted with caution.^20,21^ Register studies similar to the present one are not well-suited to show the value of adjuvant chemotherapy. Nonetheless, they do play a role in defining the need for such therapy in real-life situations.

In accordance with previous studies, the prognosis for patients in stage 0 was excellent both concerning DM and LRR, irrespective of whether they received CRT/TNT or only scRT with delay to surgery.^5,22^ The recurrence rates in these initially advanced tumors responding completely were not higher than in the early tumors in stage I operated directly. Of great interest also was the finding, albeit statistically insignificant, that the presence of RFs, i.e., either a low tumor level or a low number of examined lymph nodes meant a higher risk of DM. However, this needs future confirmation because of the low absolute number of events.

Tumor deposits, aside from (y)pT and (y)pN, had the largest impact on both LRR and DM in the present study. Previous rectal cancer studies have shown the presence of TD to be a poor prognostic marker in patients with locally advanced cancers receiving neoadjuvant treatment.^23–25^ An important finding in our study is that this holds even for individuals who did not receive pretreatment.

The distinction between intra- and extramural vascular invasion, as well as lymphovascular invasion, was not made in the SCRCR until 2017. It is our understanding that most cases of vascular invasion in the registry should be interpreted as extramural vascular invasion. Nevertheless, vascular invasion proved to be an independent marker for both DM and LRR. Similar results have previously been described in a study from the SCRCR conducted on patients in stage II.^26^

While there is a lack of consensus regarding the precise definition of perineural invasion, studies indicate a correlation with both LRR and DFS.^27,28^ We found that perineural invasion was an independent RF for DM (and DFS and OS) but not LLR.

Circumferential resection margin is associated with DM in several publications. Detering et al. analyzed 75 studies regarding the importance of CRM positivity, four of which had conducted a multivariable regression for DM and defined a positive CRM as ≤ 1 mm.^29^ They concluded that a positive CRM was a poor prognostic factor for DM (and LRR, DFS, OS). Even though we also noted a statistically significant association between CRM and DM in an unadjusted regression analysis, none were seen when adjusting for other major factors, in particular (y)pT, (y)pN, and TD in our cohort. This finding persisted when grouped by preoperative treatment. The reason for the discrepancy between the meta-analysis and the present study may be explained by the evolution of oncological treatments and the dissemination of high-quality TME surgery. Previous research within the SCRCR identified CRM+ as an independent RF for both LRR and DM. However, those studies could not adjust for TD and were somewhat constrained by missing data about vascular and perineural invasion.^30,31^

In the Dutch TME-trial, which randomized consecutive patients with resectable rectal cancer to TME alone or scRT without delay to surgery, the rates of DM after 5 years were 26% and 28%, respectively.^2^ Similar rates were seen in the Stockholm III trial with recruitment between 1998 and 2013.^32^ The DM rates of 14% and 17% in the current material is markedly lower compared with the aforementioned studies. This can at least partly be explained by improvements in the TME surgery, no use of preoperative and limited use of postoperative chemotherapy in the two trials, and the improvement in the detection of synchronous detection of metastasis (i.e., stage migration). More modern materials with data on intermediate and high-risk rectal cancers demonstrate DM rates of 27–30% at 5 years after surgery when given CRT.^33,34^ Similar patients given CRT in our material showed a DM rate of 21%. Reduced stage-specific recurrence risks also were seen between 2004 and 2019 in a recent Danish population-based study.^35^ In the study, patients at the end of the study period had similar recurrence rates as seen in our material.

Locoregional recurrence rates were reduced from approximately 20% in the decades leading up to the introduction of TME and more frequent use of preoperative RT/CRT, down to approximately 8%.^36^ The overall rate of LRR was 3% in the present study, comparable to a recent population-based study from Australia and lower than in studies describing historical rates in the SCRCR.^15,37^ The locoregional recurrence rate only exceeded 4% for patients in stage II and III with two or more RFs, which confirms the diagnostic and therapeutic advances during recent decades. Tumor level ≤ 5 cm was associated with one of the highest risks of LRR in the analysis of the whole cohort, whereas subgroup analysis only revealed an increased risk for patients without pretreatment. This finding raises the question of whether neoadjuvant therapy should be routinely given to all low tumors, regardless of clinical T-stage.

An inherent limitation to epidemiological studies of this nature is the inability to establish direct causality when correlations are found. While likely most confounding factors have been addressed through adjustments, some may still influence our results. Nevertheless, this nationwide study offers real-world insights into the current state of rectal cancer treatment in a country with high abidance to guidelines. Furthermore, despite the valuable assistance provided by quality assurance guidelines to pathologists, it is important to acknowledge the challenges. Adherence to such guidelines cannot be guaranteed, and variations between observers may impact the differentiation between, for instance, lymph node metastases and TD or vascular and perineural invasion.^38^

Given the purpose of our study, recurrence risks rather than DFS and OS were the outcomes of primary interest even if the latter are considered more relevant when describing the results of different interventions when pros and cons have to be discussed. Recurrence risks are frequently less often reliably registered in registries than, e.g., death. However, SCRCR has high coverage of both LRR and DM if follow-up exceeds 5 years, as in this study.^17^ It is mandatory to report all recurrences after they have occurred and reminders are sent to the treating hospital after 3 and 5 years. There is a potential underestimation of recurrence rates in this study attributed to the absence of competing risk analysis, a limitation compounded by the relatively low rate of autopsies conducted in Sweden. Furthermore, we observed a comparably low rate of DM around the time of reporting LRR. While many parameters within the SCRCR have been validated, the reliability of reporting synchronous metastasis remains unknown.

It is evident that some subgroups of patients still face an unacceptably high risk of DM. If adjuvant therapy, whether it consists of conventional chemotherapy or novel tailored therapies can reduce this risk should be the subject of future research. This study strengthens the notion that the final pathological stage dictates long-term outcomes in rectal cancer patients with and without neoadjuvant therapy. Echoing the proposal of others, we also advocate for tailored postoperative surveillance in selected patient groups.^35^ This selection may be aided by the risk stratification depicted in this work.

## Conclusions

Readily available variables are still robust in predicting the risk for both DM and LRR in rectal cancer. Stratification by stage, risk factors, and preoperative treatment reveal those at low or a more or less high risk of recurrence. Tumor deposits, while associated with some diagnostic difficulties, is an important risk factor for both DM and LRR. Circumferential resection margin did not prove to be an independent factor for DM or LRR and needs to be the subject of further examination in future materials. A tailored postoperative surveillance is possible in selected groups using risk stratification based on stage and risk factors.

### Supplementary Information

Below is the link to the electronic supplementary material.

Supplementary file1 (PDF 420 kb)

## Data Availability

The datasets produced and examined in the course of the current study were obtained from the Swedish Colorectal Cancer Registry after authorization by the Registry steering committee and the Swedish Ethical Review Authority. The data are not publicly available but can be obtained upon request and approval from the SCRCR and Swedish Ethical Review Authority.
