# Supplementary material for: Risk Factors for Recurrence After Surgery for Rectal Cancer in a Modern, Nationwide Population-Based Cohort
Source: Ann Surg Oncol. 2024 Jun 9;31(9):5570–84. doi: 10.1245/s10434-024-15552-x (PMC11300512; doi:10.1245/s10434-024-15552-x)
Supplement: Supplementary file 1 — Supplementary file1 (PDF 420 kb) [file 10434_2024_15552_MOESM1_ESM.pdf]

Table S1. Cox proportional hazards for OS and DFS

Characteristic	Univariable OS				Univariable DFS				Multivariable OS				Multivariable DFS			
	N events	HR	95% CI	p-value[1]	N events	HR	95% CI	p-value[1]	N events	HR	95% CI	p-value[1]	N events	HR	95% CI	p-value[1]
**Sex**	2535			**<0.001**	3137			**<0.001**	1832			**<0.001**	2308			**<0.001**
Female		Ref	Ref			Ref	Ref			Ref	Ref			Ref	Ref	
Male		1.21	1.11, 1.31			1.18	1.09, 1.26			1.27	1.15, 1.40			1.21	1.11, 1.31	
**Age,** years				**<0.001**				**<0.001**				**<0.001**				**<0.001**
<75		Ref	Ref			Ref	Ref			Ref	Ref			Ref	Ref	
≥75		2.73	2.53, 2.95			1.96	1.82, 2.10			2.12	1.91, 2.36			1.57	1.43, 1.73	
**ASA**				**<0.001**				**<0.001**				**<0.001**				**<0.001**
1		Ref	Ref			Ref	Ref			Ref	Ref			Ref	Ref	
2		1.77	1.55, 2.02			1.45	1.30, 1.62			1.42	1.21, 1.66			1.24	1.09, 1.42	
3		3.85	3.36, 4.43			2.53	2.25, 2.84			2.52	2.12, 2.99			1.80	1.56, 2.08	
4		6.88	5.23, 9.05			4.01	3.09, 5.21			4.46	3.29, 6.06			2.95	2.21, 3.94	
**Surgery**				**<0.001**				**<0.001**								
AR		Ref	Ref			Ref	Ref									
APR		1.81	1.66, 1.97			1.59	1.47, 1.71									
Hartmann		3.19	2.86, 3.56			2.33	2.11, 2.58									
**Tumour Level (cm)**				**<0.001**				**<0.001**				**<0.001**				**<0.001**
11-15		Ref	Ref			Ref	Ref			Ref	Ref			Ref	Ref	
6-10		1.05	0.95, 1.16			1.08	0.99, 1.18			1.12	0.99, 1.25			1.17	1.05, 1.30	
0-5		1.28	1.16, 1.42			1.25	1.14, 1.37			1.41	1.23, 1.60			1.36	1.21, 1.53	
**AJCC Stage**				**<0.001**				**<0.001**								
I		Ref	Ref			Ref	Ref									
0		0.81	0.59, 1.11			0.84	0.63, 1.11									
II		1.60	1.43, 1.78			1.74	1.58, 1.92									
III		2.48	2.24, 2.75			2.80	2.55, 3.07									
**pT**				**<0.001**				**<0.001**				**<0.001**				**<0.001**
T1		Ref	Ref			Ref	Ref			Ref	Ref			Ref	Ref	
T0		0.96	0.70, 1.32			1.00	0.75, 1.35			1.42	0.79, 2.53			1.45	0.84, 2.49	
T2		1.21	1.00, 1.46			1.29	1.09, 1.53			1.10	0.89, 1.37			1.23	1.01, 1.51	
T3ab		1.79	1.49, 2.14			2.07	1.75, 2.44			1.45	1.17, 1.80			1.76	1.44, 2.15	
T3cd		2.58	2.13, 3.12			3.10	2.60, 3.69			1.87	1.48, 2.36			2.26	1.82, 2.80	
T3 unknown		2.20	1.78, 2.71			2.66	2.19, 3.23			1.61	1.23, 2.10			2.01	1.58, 2.57	
T4a		3.41	2.66, 4.37			3.88	3.10, 4.87			2.37	1.75, 3.20			2.87	2.18, 3.77	
T4b		3.50	2.71, 4.52			3.64	2.88, 4.62			2.53	1.85, 3.47			2.59	1.93, 3.46	
T4 unknown		4.75	3.16, 7.14			6.67	4.63, 9.63			3.59	2.21, 5.84			5.95	3.87, 9.14	
**pN**				**<0.001**				**<0.001**				**<0.001**				**<0.001**
N0		Ref	Ref			Ref	Ref			Ref	Ref			Ref	Ref	
N1a		1.50	1.33, 1.69			1.68	1.52, 1.87			1.37	1.19, 1.58			1.50	1.32, 1.70	
N1b		1.83	1.63, 2.06			1.97	1.77, 2.19			1.71	1.47, 1.98			1.73	1.52, 1.98	
N2a		2.30	2.01, 2.64			2.34	2.07, 2.66			2.18	1.82, 2.61			1.89	1.60, 2.22	
N2b		3.10	2.70, 3.56			3.41	3.01, 3.87			2.58	2.13, 3.11			2.41	2.03, 2.86	
**Lymph Node Yield**				**<0.001**				**<0.001**				**<0.001**				**<0.001**
≥12		Ref	Ref			Ref	Ref			Ref	Ref			Ref	Ref	
<12		1.32	1.19, 1.47			1.26	1.14, 1.38			1.36	1.19, 1.54			1.39	1.24, 1.56	
**Tumor Deposit**				**<0.001**				**<0.001**				**<0.001**				**<0.001**
No		Ref	Ref			Ref	Ref			Ref	Ref			Ref	Ref	
Yes		2.15	1.94, 2.38			2.42	2.21, 2.64			1.47	1.30, 1.67			1.48	1.33, 1.65	
**Perforation**				**<0.001**				**<0.001**				0.47				0.14
No perforation		Ref	Ref			Ref	Ref			Ref	Ref			Ref	Ref	
Perf not near tumour		1.35	1.05, 1.73			1.21	0.96, 1.53			0.97	0.70, 1.33			0.84	0.63, 1.13	
Perf near tumour		1.58	1.23, 2.03			1.57	1.24, 1.97			1.20	0.90, 1.61			1.24	0.96, 1.62	
**Differentiation**				**<0.001**				**<0.001**				0.24				0.68
High/moderate		Ref	Ref			Ref	Ref			Ref	Ref			Ref	Ref	
Poor		1.39	1.24, 1.55			1.36	1.23, 1.50			1.08	0.95, 1.24			1.03	0.91, 1.16	
**CRM (mm)**				**<0.001**				**<0.001**				0.066				**0.007**
≥ 2		Ref	Ref			Ref	Ref			Ref	Ref			Ref	Ref	
1.1-1.9		0.96	0.83, 1.10			0.93	0.82, 1.06			1.03	0.84, 1.28			1.05	0.87, 1.26	
≤1		2.02	1.79, 2.29			2.20	1.97, 2.46			1.22	1.04, 1.43			1.27	1.10, 1.46	
**Mucinous**				**<0.001**				**<0.001**				0.34				0.96
No		Ref	Ref			Ref	Ref			Ref	Ref			Ref	Ref	
Yes		1.39	1.23, 1.56			1.33	1.20, 1.48			1.08	0.93, 1.26			1.00	0.87, 1.15	
**Perineural Invasion**				**<0.001**				**<0.001**				**<0.001**				**<0.001**
No		Ref	Ref			Ref	Ref			Ref	Ref			Ref	Ref	
Yes		2.18	2.00, 2.39			2.33	2.14, 2.52			1.34	1.19, 1.52			1.33	1.19, 1.48	
**Vascular Invasion**				**<0.001**				**<0.001**				0.21				**<0.001**
No		Ref	Ref			Ref	Ref			Ref	Ref			Ref	Ref	
Yes		1.77	1.63, 1.93			1.92	1.78, 2.07			1.08	0.96, 1.20			1.19	1.08, 1.31	
**Neoadjuvant treatment**				**<0.001**				**<0.001**				**<0.001**				**<0.001**
Surgery only		Ref	Ref			Ref	Ref			Ref	Ref			Ref	Ref	
scRT		0.85	0.77, 0.94			0.95	0.87, 1.03			0.83	0.74, 0.94			0.86	0.77, 0.96	
scRT-delay		1.49	1.33, 1.68			1.47	1.32, 1.64			1.12	0.98, 1.29			1.17	1.03, 1.33	
CRT		0.90	0.80, 1.01			1.05	0.94, 1.17			1.11	0.94, 1.30			1.12	0.97, 1.28	
TNT		0.76	0.58, 0.99			1.06	0.86, 1.31			0.97	0.70, 1.34			1.21	0.94, 1.56	
**Adjuvant treatment**				**<0.001**				0.25				**<0.001**				**<0.001**
None		Ref	Ref			Ref	Ref			Ref	Ref			Ref	Ref	
Only FU		0.82	0.72, 0.93			1.04	0.93, 1.16			0.63	0.54, 0.74			0.73	0.64, 0.84	
FU + Oxaliplatin		0.83	0.73, 0.94			1.09	0.98, 1.22			0.61	0.51, 0.74			0.68	0.58, 0.80	

HR, Hazard Ratio, CI, Confidence Interval. OS, overall survival. DFS, disease-free survival. Ref, reference. ASA, American Society of Anethesiologists Physical Status.
AJCC, American Joint Committee on Cancer. Perf, perforation. AR, Anterior resection. APR, Abdominoperineal resection. CRM, circumferential resection margin.
scRT, short-course radiotherapy. scRT-delay, scRT with delay to surgery. CRT, chemoradiotherapy. TNT, total neoadjuvant therapy. FU, fluoropyrimidine. 1Pearson's Chi-squared test.

Table S2a. Cox proportional hazards for DM stratified by pre-treatment

Distant metastasis (DM)

Univariable

	Surgery only				scRT				scRT-delay				CRT/TNT				CRT only			
**Characteristic**	**N events**	**HR**	**95% CI**	**p-value[1]**	**N events**	**HR**	**95% CI**	**p-value[1]**	**N events**	**HR**	**95% CI**	**p-value[1]**	**N events**	**HR**	**95% CI**	**p-value[1]**	**N events**	**HR**	**95% CI**	**p-value[1]**
**Sex**	423			**0.038**	478			0.97	233			0.97	391			0.13	322			0.15
Female		Ref				Ref				Ref				Ref				Ref		
Male		1.23	1.01, 1.50			1.00	0.83, 1.20			1.01	0.77, 1.31			1.17	0.95, 1.44			1.18	0.94, 1.48	
**Age**				0.70				0.93				0.34				0.60				0.65
<75		Ref				Ref				Ref				Ref				Ref		
≥75		1.04	0.85, 1.27			1.01	0.82, 1.24			1.13	0.88, 1.46			0.91	0.64, 1.30			0.92	0.63, 1.34	
**ASA**				**0.032**				0.078				0.33				0.41				0.53
1		Ref				Ref				Ref				Ref				Ref		
2		1.05	0.80, 1.39			1.03	0.83, 1.29			1.04	0.68, 1.59			1.01	0.79, 1.30			1.09	0.83, 1.44	
3		1.40	1.04, 1.88			1.09	0.81, 1.45			0.96	0.61, 1.51			1.18	0.86, 1.61			1.21	0.85, 1.72	
4		1.77	0.81, 3.85			0.00	0.00, Inf			1.96	0.92, 4.19			0.00	0.00, Inf			0.00	0.00, Inf	
**Surgery**				**<0.001**				0.77				0.093				0.069				0.10
AR		Ref				Ref				Ref				Ref				Ref		
APR		1.85	1.50, 2.30			1.05	0.88, 1.27			1.37	1.03, 1.82			1.27	1.03, 1.56			1.25	0.99, 1.58	
Hartmann		1.31	0.99, 1.73			0.95	0.66, 1.36			1.25	0.82, 1.91			1.27	0.86, 1.87			1.40	0.92, 2.12	
**Tumour Level (cm)**				**<0.001**				0.69				0.29				0.061				**0.019**
11-15		Ref				Ref				Ref				Ref				Ref		
6-10		0.97	0.78, 1.20			0.95	0.74, 1.22			0.96	0.67, 1.39			1.41	1.05, 1.89			1.60	1.14, 2.24	
0-5		1.66	1.28, 2.15			1.03	0.80, 1.34			1.20	0.84, 1.72			1.32	0.98, 1.78			1.42	1.01, 2.01	
**AJCC Stage**				**<0.001**				**<0.001**				**<0.001**				**<0.001**				**<0.001**
I		Ref				Ref				Ref				Ref				Ref		
0		NA	NA			3.19	0.78, 13.1			0.58	0.20, 1.67			0.82	0.43, 1.56			1.16	0.59, 2.31	
II		2.98	2.22, 4.00			1.80	1.31, 2.47			3.16	2.05, 4.87			2.13	1.45, 3.14			2.25	1.46, 3.49	
III		5.44	4.15, 7.16			4.82	3.64, 6.40			5.33	3.51, 8.12			5.37	3.71, 7.77			5.41	3.57, 8.20	
**pT**				**<0.001**				**<0.001**				**<0.001**				**<0.001**				**<0.001**
T0-2		Ref				Ref				Ref				Ref				Ref		
T3		3.25	2.58, 4.08			2.22	1.79, 2.75			3.34	2.41, 4.63			2.67	2.08, 3.43			2.55	1.94, 3.36	
T4		6.72	4.85, 9.32			5.40	3.73, 7.81			5.13	3.24, 8.12			2.84	1.99, 4.06			2.87	1.94, 4.25	
**pN**				**<0.001**				**<0.001**				**<0.001**				**<0.001**				**<0.001**
N0		Ref				Ref				Ref				Ref				Ref		
N1a		2.11	1.59, 2.79			2.08	1.56, 2.78			2.27	1.55, 3.33			2.98	2.26, 3.94			2.91	2.14, 3.96	
N1b		2.81	2.12, 3.74			3.19	2.48, 4.10			2.56	1.75, 3.74			3.22	2.42, 4.28			2.87	2.08, 3.95	
N2a		3.81	2.76, 5.26			2.96	2.18, 4.00			3.14	1.97, 5.00			4.40	3.20, 6.05			4.26	3.01, 6.02	
N2b		6.46	4.73, 8.81			6.75	5.22, 8.72			4.56	2.95, 7.03			4.49	3.12, 6.48			4.23	2.86, 6.26	
**Lymph Node Yield**				**0.034**				**0.014**				0.78				0.69				0.32
≥12		Ref				Ref				Ref				Ref				Ref		
<12		1.36	1.03, 1.79			1.37	1.07, 1.74			1.05	0.73, 1.53			1.05	0.82, 1.35			1.15	0.88, 1.52	
**Tumor Deposit**				**<0.001**				**<0.001**				**<0.001**				**<0.001**				**<0.001**
No		Ref				Ref				Ref				Ref				Ref		
Yes		4.30	3.42, 5.40			3.14	2.56, 3.86			2.48	1.81, 3.38			3.22	2.58, 4.01			3.17	2.49, 4.04	
**Perforation**				**0.007**				0.59				0.41				0.26				0.10
No perforation		Ref				Ref				Ref				Ref				Ref		
Perf not near tumor		0.88	0.42, 1.86			0.84	0.40, 1.78			0.84	0.35, 2.04			0.86	0.41, 1.81			1.06	0.50, 2.24	
Perf near tumor		2.41	1.48, 3.91			1.38	0.71, 2.67			1.69	0.80, 3.58			1.69	0.93, 3.08			2.16	1.15, 4.05	
**Differentiation**				**<0.001**				**<0.001**				**0.011**				**0.017**				**0.035**
High/moderate		Ref				Ref				Ref				Ref				Ref		
Poor		1.68	1.29, 2.18			1.57	1.27, 1.95			1.65	1.14, 2.38			1.46	1.09, 1.97			1.46	1.04, 2.04	
**CRM (mm)**				**<0.001**				**<0.001**				**<0.001**				**<0.001**				**<0.001**
≥ 2		Ref				Ref				Ref				Ref				Ref		
1.1-1.9		0.91	0.62, 1.33			1.40	0.98, 2.00			0.66	0.40, 1.08			0.68	0.49, 0.94			0.86	0.60, 1.21	
≤1		2.92	2.19, 3.90			2.39	1.80, 3.16			2.48	1.70, 3.62			1.66	1.24, 2.21			1.87	1.38, 2.52	
**Mucinous**				**<0.001**				**0.023**				**0.006**				0.51				0.58
No		Ref				Ref				Ref				Ref				Ref		
Yes		1.84	1.39, 2.43			1.39	1.06, 1.83			1.72	1.20, 2.48			1.11	0.82, 1.49			1.10	0.79, 1.54	
**Perineural Invasion**				**<0.001**				**<0.001**				**<0.001**				**<0.001**				**<0.001**
No		Ref				Ref				Ref				Ref				Ref		
Yes		3.79	3.07, 4.67			2.85	2.36, 3.44			2.68	2.03, 3.54			2.94	2.38, 3.63			2.98	2.36, 3.77	
**Vascular Invasion**				**<0.001**				**<0.001**				**<0.001**				**<0.001**				**<0.001**
No		Ref				Ref				Ref				Ref				Ref		
Yes		2.53	2.09, 3.08			2.51	2.09, 3.01			2.34	1.77, 3.09			2.68	2.18, 3.31			2.58	2.05, 3.25	
**Adjuvant Treatment**				**<0.001**				**<0.001**				**0.007**				**<0.001**				**<0.001**
None		Ref				Ref				Ref				Ref				Ref		
Only FU		1.79	1.38, 2.32			1.97	1.57, 2.49			1.18	0.75, 1.85			1.88	1.46, 2.43			2.08	1.59, 2.73	
FU + Oxaliplatin		1.94	1.46, 2.57			2.49	1.99, 3.11			2.36	1.45, 3.82			1.74	1.37, 2.22			1.93	1.49, 2.50	

Multivariable

	Surgery only				scRT				scRT-delay				CRT/TNT				CRT only			
**Characteristic**	**N events**	**HR**	**95% CI**	**p-value[1]**	**N events**	**HR**	**95% CI**	**p-value[1]**	**N events**	**HR**	**95% CI**	**p-value[1]**	**N events**	**HR**	**95% CI**	**p-value[1]**	**N events**	**HR**	**95% CI**	**p-value[1]**
**Sex**	337			0.072	356			0.91	191			0.53	271			0.38	218			0.50
Female		Ref				Ref				Ref				Ref				Ref		
Male		1.23	0.98, 1.55			0.99	0.79, 1.23			0.91	0.68, 1.22			1.14	0.85, 1.52			1.11	0.82, 1.51	
**Age**				0.23				0.38				0.48				0.63				0.60
<75		Ref				Ref				Ref				Ref				Ref		
≥75		0.86	0.67, 1.10			0.89	0.67, 1.16			0.89	0.66, 1.22			1.13	0.70, 1.83			1.14	0.70, 1.87	
**ASA**				0.89				0.16				0.093				0.72				0.68
1		Ref				Ref				Ref				Ref				Ref		
2		1.06	0.76, 1.47			0.94	0.73, 1.22			1.14	0.71, 1.85			0.96	0.69, 1.34			0.93	0.66, 1.32	
3		1.15	0.79, 1.68			0.88	0.62, 1.25			0.91	0.54, 1.52			0.90	0.58, 1.39			0.86	0.54, 1.39	
4		1.09	0.46, 2.59			0.00	0.00, Inf			2.39	1.07, 5.35			0.00	0.00, Inf			0.00	0.00, Inf	
**Surgery**																				
AR																				
APR																				
Hartmann																				
**Tumour Level (cm)**				**0.014**				0.38				**0.027**				0.14				0.19
11-15		Ref				Ref				Ref				Ref				Ref		
6-10		1.17	0.91, 1.50			1.03	0.77, 1.37			1.21	0.79, 1.85			1.47	0.99, 2.16			1.46	0.96, 2.22	
0-5		1.63	1.18, 2.24			1.20	0.88, 1.63			1.70	1.10, 2.64			1.33	0.88, 2.01			1.30	0.84, 2.02	
**AJCC Stage**																				
I																				
0																				
II																				
III																				
**pT**				**<0.001**				**<0.001**				**<0.001**				**0.030**				**0.010**
T0-2		Ref				Ref				Ref				Ref				Ref		
T3		2.36	1.78, 3.13			1.65	1.25, 2.17			2.46	1.64, 3.69			1.58	1.08, 2.31			1.82	1.21, 2.74	
T4		3.89	2.56, 5.92			2.91	1.78, 4.74			3.51	1.98, 6.20			1.85	1.10, 3.14			1.90	1.08, 3.37	
**pN**				**<0.001**				**<0.001**				**<0.001**				**<0.001**				**<0.001**
N0		Ref				Ref				Ref				Ref				Ref		
N1a		1.69	1.22, 2.35			1.80	1.27, 2.55			1.81	1.17, 2.80			2.10	1.44, 3.06			1.98	1.32, 2.96	
N1b		1.78	1.24, 2.54			2.85	2.07, 3.93			2.01	1.29, 3.13			2.16	1.43, 3.27			2.16	1.39, 3.35	
N2a		2.24	1.47, 3.42			2.24	1.49, 3.39			2.50	1.45, 4.33			3.16	2.03, 4.92			3.85	2.43, 6.12	
N2b		3.47	2.28, 5.27			5.06	3.47, 7.40			2.52	1.42, 4.45			2.45	1.43, 4.20			2.68	1.50, 4.77	
**Lymph Node Yield**				**0.018**				**<0.001**				0.076				0.18				0.13
≥12		Ref				Ref				Ref				Ref				Ref		
<12		1.54	1.09, 2.16			1.79	1.31, 2.44			1.48	0.98, 2.25			1.29	0.90, 1.84			1.36	0.93, 2.00	
**Tumor Deposit**				**<0.001**				**<0.001**				0.29				**0.001**				**<0.001**
No		Ref				Ref				Ref				Ref				Ref		
Yes		1.79	1.34, 2.38			1.77	1.37, 2.28			1.23	0.84, 1.81			1.73	1.25, 2.39			1.83	1.31, 2.56	
**Perforation**				0.12				0.51				0.089				0.94				0.92
No perforation		Ref				Ref				Ref				Ref				Ref		
Perf not near tumor		0.42	0.16, 1.15			1.04	0.49, 2.21			0.29	0.07, 1.23			0.98	0.36, 2.68			0.82	0.26, 2.60	
Perf near tumor		1.19	0.66, 2.16			1.58	0.76, 3.27			1.57	0.57, 4.32			1.15	0.54, 2.44			0.90	0.38, 2.15	
**Differentiation**				0.077				0.51				0.64				0.54				0.67
High/moderate		Ref				Ref				Ref				Ref				Ref		
Poor		0.74	0.53, 1.04			1.09	0.84, 1.41			1.11	0.72, 1.70			0.88	0.58, 1.33			0.91	0.59, 1.40	
**CRM (mm)**				0.82				0.56				0.35				0.63				0.53
≥ 2		Ref				Ref				Ref				Ref				Ref		
1.1-1.9		0.90	0.55, 1.47			1.15	0.72, 1.82			1.07	0.55, 2.07			0.94	0.54, 1.63			0.87	0.48, 1.60	
≤1		1.08	0.74, 1.59			1.19	0.84, 1.69			1.42	0.90, 2.24			1.20	0.80, 1.80			1.23	0.81, 1.89	
**Mucinous**				0.13				0.74				**0.041**				0.068				0.10
No		Ref				Ref				Ref				Ref				Ref		
Yes		1.32	0.93, 1.86			0.94	0.67, 1.33			1.60	1.04, 2.47			0.64	0.39, 1.06			0.66	0.39, 1.10	
**Perineural Invasion**				**<0.001**				**0.037**				0.14				**0.017**				**0.010**
No		Ref				Ref				Ref				Ref				Ref		
Yes		1.69	1.27, 2.24			1.30	1.02, 1.67			1.32	0.92, 1.90			1.51	1.08, 2.10			1.59	1.12, 2.26	
**Vascular Invasion**				0.15				0.10				0.083				0.14				0.24
No		Ref				Ref				Ref				Ref				Ref		
Yes		1.22	0.93, 1.59			1.23	0.96, 1.56			1.38	0.96, 1.99			1.28	0.93, 1.77			1.23	0.87, 1.73	
**Adjuvant Treatment**				0.10				**0.025**				**0.004**				0.081				**0.043**
None		Ref				Ref				Ref				Ref				Ref		
Only FU		0.92	0.67, 1.26			0.96	0.72, 1.30			0.40	0.22, 0.73			1.37	0.98, 1.90			1.32	0.93, 1.88	
FU + Oxaliplatin		0.66	0.45, 0.98			0.64	0.45, 0.90			0.87	0.47, 1.63			0.93	0.65, 1.33			0.79	0.54, 1.16	

HR, Hazard Ratio, CI, Confidence Interval. DM, distant metastasis. Ref, reference. ASA, American Society of Anethesiologists Physical Status. AJCC, American Joint Committee on Cancer. Perf, perforation.
AR, Anterior resection. APR, Abdominoperineal resection. CRM, circumferential resection margin. scRT, short-course radiotherapy. scRT-delay, scRT with delay to surgery.
CRT, chemoradiotherapy. TNT, total neoadjuvant therapy. FU, fluoropyrimidine. [1]Pearson's Chi-squared test.

Table S2b. Cox proportional hazards for LRR stratified by pre-treatment

Locoregional recurrence (LRR)

Univariable

Characteristic	Surgery only				scRT				scRT-delay				CRT/TNT				CRT only			
	N events	HR	95% CI	p-value[1]	N events	HR	95% CI	p-value[1]	N events	HR	95% CI	p-value[1]	N events	HR	95% CI	p-value[1]	N events	HR	95% CI	p-value[1]
**Sex**	106			0.26	59			0.80	42			0.15	75			0.58	61			0.28
Female		Ref				Ref				Ref				Ref				Ref		
Male		1.25	0.84, 1.86			0.93	0.56, 1.57			0.64	0.35, 1.17			1.14	0.71, 1.83			1.34	0.78, 2.29	
**Age**				0.074				0.97				0.28				0.41				0.18
<75		Ref	Ref			Ref				Ref				Ref				Ref		
≥75		1.42	0.97, 2.09			0.99	0.54, 1.80			1.39	0.76, 2.55			0.70	0.28, 1.73			0.49	0.15, 1.56	
**ASA**				0.067				0.41				0.47				0.47				0.51
1		Ref				Ref				Ref				Ref				Ref		
2		1.01	0.57, 1.81			1.68	0.81, 3.47			1.11	0.42, 2.90			1.21	0.69, 2.13			1.14	0.62, 2.10	
3		1.73	0.95, 3.15			1.61	0.65, 3.95			0.83	0.29, 2.38			0.72	0.31, 1.68			0.63	0.24, 1.64	
4		2.20	0.50, 9.62			0.00	0.00, Inf			0.00	0.00, Inf			0.00	0.00, Inf			0.00	0.00, Inf	
**Surgery**				**<0.001**				0.21				0.33				**0.008**				**0.003**
AR		Ref				Ref				Ref				Ref				Ref		
APR		2.32	1.49, 3.61			1.08	0.62, 1.87			1.38	0.72, 2.65			2.12	1.28, 3.51			2.57	1.43, 4.63	
Hartmann		2.61	1.60, 4.24			2.15	0.97, 4.75			0.65	0.19, 2.26			1.21	0.42, 3.53			1.30	0.38, 4.48	
**Tumour Level (cm)**				**0.014**				0.58				0.60				**0.050**				0.068
11-15		Ref				Ref				Ref				Ref				Ref		
6-10		1.32	0.85, 2.05			1.31	0.60, 2.89			1.36	0.54, 3.45			2.26	1.00, 5.11			2.52	0.97, 6.54	
0-5		2.22	1.33, 3.72			1.52	0.68, 3.39			1.58	0.63, 3.98			2.47	1.09, 5.59			2.64	1.02, 6.86	
**AJCC Stage**				**<0.001**				**<0.001**				**<0.001**				**<0.001**				**<0.001**
I		Ref				Ref				Ref				Ref				Ref		
0		0.00	0.00, Inf			0.00	0.00, Inf			0.00	0.00, Inf			2.80	0.63, 12.5			1.66	0.28, 9.95	
II		2.14	1.26, 3.64			1.48	0.61, 3.58			1.57	0.69, 3.59			4.83	1.45, 16.0			4.24	1.26, 14.3	
III		3.34	2.04, 5.48			3.90	1.81, 8.42			2.29	1.03, 5.11			8.53	2.61, 27.9			6.34	1.91, 21.1	
**pT**				**<0.001**				**<0.001**				**<0.001**				**<0.001**				**<0.001**
T0-2		Ref				Ref				Ref				Ref				Ref		
T3		2.66	1.67, 4.22			3.24	1.63, 6.42			2.16	1.03, 4.54			3.69	1.93, 7.08			4.93	2.23, 10.9	
T4		9.76	5.53, 17.2			4.22	1.16, 15.3			6.54	2.66, 16.1			4.52	1.96, 10.4			5.17	1.87, 14.3	
**pN**				**<0.001**				**<0.001**				0.28				**<0.001**				**0.002**
N0		Ref				Ref				Ref				Ref				Ref		
N1a		1.53	0.83, 2.82			3.01	1.45, 6.25			2.16	0.93, 5.01			2.47	1.30, 4.69			2.22	1.07, 4.58	
N1b		2.61	1.49, 4.58			2.23	0.99, 5.04			1.94	0.79, 4.76			1.93	0.92, 4.03			1.71	0.74, 3.93	
N2a		2.64	1.30, 5.36			1.32	0.39, 4.44			1.35	0.32, 5.70			4.35	2.20, 8.61			3.99	1.88, 8.49	
N2b		5.59	2.98, 10.5			8.52	4.39, 16.5			2.37	0.71, 7.88			4.04	1.79, 9.15			3.69	1.52, 8.95	
**Lymph Node Yield**				0.80				**0.008**				0.35				0.93				0.60
≥12		Ref				Ref				Ref				Ref				Ref		
<12		1.08	0.59, 1.97			2.36	1.31, 4.25			1.47	0.68, 3.17			1.03	0.57, 1.84			0.83	0.41, 1.69	
**Tumor Deposit**				**<0.001**				**<0.001**				**<0.001**				**0.007**				**0.032**
No		Ref				Ref				Ref				Ref				Ref		
Yes		4.75	3.03, 7.45			3.29	1.85, 5.84			3.44	1.78, 6.64			2.21	1.28, 3.81			2.04	1.10, 3.77	
**Perforation**				**0.025**				0.36				**0.018**				**0.007**				**0.001**
No perforation		Ref				Ref				Ref				Ref				Ref		
Perf not near tumor		2.70	1.10, 6.64			0.00	0.00, Inf			2.14	0.52, 8.92			0.68	0.09, 4.90			0.86	0.12, 6.22	
Perf near tumor		2.92	1.19, 7.18			1.16	0.16, 8.38			6.00	2.13, 16.9			5.25	2.27, 12.1			7.41	3.18, 17.3	
**Differentiation**				**<0.001**				0.11				0.051				**<0.001**				**0.002**
High/moderate		Ref				Ref				Ref				Ref				Ref		
Poor		2.55	1.60, 4.06			1.69	0.92, 3.12			2.34	1.07, 5.10			3.27	1.84, 5.80			3.11	1.63, 5.94	
**CRM (mm)**				**<0.001**				**0.002**				**<0.001**				**<0.001**				**<0.001**
≥ 2		Ref				Ref				Ref				Ref				Ref		
1.1-1.9		0.85	0.37, 1.96			2.25	0.96, 5.29			0.26	0.04, 1.90			1.48	0.78, 2.82			1.83	0.89, 3.74	
≤1		6.00	3.79, 9.51			3.72	1.87, 7.41			6.48	3.35, 12.5			5.11	3.06, 8.54			5.77	3.30, 10.1	
**Mucinous**				**0.007**				0.53				**0.001**				0.28				0.11
No		Ref				Ref				Ref				Ref				Ref		
Yes		2.20	1.31, 3.71			1.30	0.59, 2.88			3.51	1.76, 7.01			1.42	0.77, 2.65			1.76	0.91, 3.39	
**Perineural Invasion**				**<0.001**				**0.006**				**0.005**				**<0.001**				**<0.001**
No		Ref				Ref				Ref				Ref				Ref		
Yes		3.71	2.42, 5.68			2.22	1.29, 3.82			2.73	1.43, 5.24			3.51	2.19, 5.65			3.08	1.81, 5.24	
**Vascular Invasion**				**<0.001**				**<0.001**				0.084				**<0.001**				**<0.001**
No		Ref				Ref				Ref				Ref				Ref		
Yes		2.78	1.88, 4.11			3.05	1.82, 5.11			1.87	0.95, 3.68			3.52	2.21, 5.60			3.41	2.03, 5.70	
**Adjuvant Treatment**				0.61				0.11				**0.021**				0.11				0.065
None		Ref				Ref				Ref				Ref				Ref		
Only FU		1.02	0.56, 1.88			1.27	0.61, 2.64			3.26	1.55, 6.84			1.24	0.65, 2.35			1.37	0.70, 2.70	
FU + Oxaliplatin		1.38	0.75, 2.52			2.05	1.09, 3.86			1.41	0.34, 5.90			1.80	1.06, 3.04			1.99	1.13, 3.51	

Multivariable

Characteristic	Surgery only				scRT				scRT-delay				CRT/TNT				CRT only			
	N events	HR	95% CI	p-value[1]	N events	HR	95% CI	p-value[1]	N events	HR	95% CI	p-value[1]	N events	HR	95% CI	p-value[1]	N events	HR	95% CI	p-value[1]
**Sex**	78			**0.022**				0.31	34			0.81	45			0.78	36			0.47
Female		Ref				Ref				Ref				Ref				Ref		
Male		1.78	1.07, 2.97			0.72	0.38, 1.35			0.91	0.43, 1.93			1.09	0.59, 2.04			1.31	0.63, 2.71	
**Age**				0.67				0.86				0.39				0.88				0.87
<75		Ref				Ref				Ref				Ref				Ref		
≥75		1.12	0.66, 1.90			1.07	0.51, 2.23			1.40	0.65, 3.05			1.10	0.32, 3.77			1.14	0.25, 5.11	
**ASA**				0.34				0.80				0.10				0.71				0.57
1		Ref				Ref				Ref				Ref				Ref		
2		0.73	0.37, 1.46			1.33	0.57, 3.08			1.60	0.45, 5.68			0.91	0.45, 1.84			0.87	0.40, 1.90	
3		1.16	0.55, 2.45			1.31	0.47, 3.68			1.00	0.24, 4.12			0.56	0.19, 1.65			0.42	0.11, 1.61	
4		0.66	0.08, 5.28			0.00	0.00, Inf			0.00	0.00, Inf			0.00	0.00, Inf			0.00	0.00, Inf	
**Surgery**																				
AR																				
APR																				
Hartmann																				
**Tumour Level (cm)**				**0.009**				0.67				0.22				0.22				0.16
11-15		Ref				Ref				Ref				Ref				Ref		
6-10		1.57	0.92, 2.71			1.28	0.52, 3.15			1.82	0.58, 5.66			1.74	0.68, 4.51			2.33	0.74, 7.35	
0-5		2.71	1.45, 5.06			1.52	0.59, 3.91			2.64	0.82, 8.49			2.20	0.86, 5.59			2.71	0.87, 8.42	
**AJCC Stage**																				
I																				
0																				
II																				
III																				
**pT**				**0.001**				**0.027**				0.43				0.11				0.052
T0-2		Ref				Ref				Ref				Ref				Ref		
T3		2.17	1.19, 3.93			3.15	1.26, 7.92			1.73	0.66, 4.51			2.70	0.99, 7.37			3.38	1.11, 10.3	
T4		4.78	2.10, 10.9			3.06	0.53, 17.7			2.13	0.60, 7.55			2.76	0.79, 9.64			2.27	0.51, 10.1	
**pN**				0.62				**<0.001**				0.89				0.41				0.80
N0		Ref				Ref				Ref				Ref				Ref		
N1a		1.04	0.51, 2.12			3.94	1.66, 9.37			1.22	0.45, 3.30			1.38	0.57, 3.30			1.22	0.46, 3.24	
N1b		1.44	0.69, 3.00			1.09	0.30, 3.99			0.66	0.19, 2.37			1.32	0.49, 3.55			1.27	0.42, 3.81	
N2a		1.83	0.79, 4.20			1.91	0.50, 7.31			0.72	0.15, 3.44			2.76	1.05, 7.25			2.15	0.69, 6.69	
N2b		0.99	0.36, 2.72			9.14	3.27, 25.5			0.70	0.17, 2.89			1.88	0.56, 6.30			1.13	0.28, 4.58	
**Lymph Node Yield**				0.61				**0.017**				0.51				0.49				0.76
≥12		Ref				Ref				Ref				Ref				Ref		
<12		0.82	0.37, 1.79			2.91	1.28, 6.63			1.39	0.53, 3.60			1.32	0.62, 2.82			0.87	0.34, 2.23	
**Tumor Deposit**				**0.008**				0.13				0.059				0.67				0.69
No		Ref				Ref				Ref				Ref				Ref		
Yes		2.27	1.26, 4.09			1.75	0.85, 3.57			2.33	0.99, 5.47			0.85	0.40, 1.79			0.84	0.36, 1.99	
**Perforation**				0.18				0.24				0.15				**0.025**				**0.001**
No perforation		Ref				Ref				Ref				Ref				Ref		
Perf not near tumor		1.85	0.65, 5.31			0.00	0.00, Inf			3.20	0.68, 15.0			0.92	0.12, 7.25			1.75	0.23, 13.5	
Perf near tumor		2.35	0.91, 6.12			0.00	0.00, Inf			4.35	0.86, 22.0			4.74	1.76, 12.8			9.30	3.40, 25.5	
**Differentiation**				0.27				0.52				0.40				0.22				0.27
High/moderate		Ref				Ref				Ref				Ref				Ref		
Poor		1.41	0.77, 2.60			1.28	0.62, 2.65			1.49	0.60, 3.70			1.62	0.76, 3.43			1.70	0.69, 4.22	
**CRM (mm)**				**0.031**				0.094				**0.001**				**0.009**				**0.002**
≥ 2		Ref				Ref				Ref				Ref				Ref		
1.1-1.9		1.17	0.42, 3.28			2.86	1.05, 7.74			0.00	0.00, Inf			1.52	0.50, 4.59			2.44	0.77, 7.72	
≤1		2.53	1.32, 4.87			1.96	0.80, 4.81			3.89	1.76, 8.60			3.31	1.61, 6.80			4.41	1.96, 9.90	
**Mucinous**				0.55				0.45				**0.002**				0.48				0.71
No		Ref				Ref				Ref				Ref				Ref		
Yes		1.23	0.63, 2.40			1.43	0.58, 3.49			4.34	1.80, 10.5			0.71	0.27, 1.87			0.81	0.27, 2.47	
**Perineural Invasion**				0.35				0.33				0.81				0.22				0.61
No		Ref				Ref				Ref				Ref				Ref		
Yes		1.33	0.73, 2.43			0.69	0.32, 1.47			1.12	0.46, 2.74			1.59	0.77, 3.27			1.25	0.54, 2.91	
**Vascular Invasion**				0.57				0.11				0.31				0.22				0.32
No		Ref				Ref				Ref				Ref				Ref		
Yes		1.18	0.67, 2.05			1.77	0.88, 3.53			1.59	0.66, 3.85			1.57	0.77, 3.18			1.52	0.68, 3.42	
**Adjuvant Treatment**				0.41				0.21				0.41				0.51				0.47
None		Ref				Ref				Ref				Ref				Ref		
Only FU		0.62	0.30, 1.29			0.50	0.19, 1.30			1.99	0.74, 5.35			0.69	0.28, 1.72			0.80	0.30, 2.11	
FU + Oxaliplatin		0.82	0.37, 1.84			0.47	0.17, 1.30			1.56	0.30, 7.98			1.22	0.58, 2.57			1.45	0.64, 3.27	

HR, Hazard Ratio, CI, Confidence Interval LRR, Locoregional recurrence. Ref, reference
ASA, American Society of Anethesiologists Physical Status. AJCC, American Joint Committee on Cancer. Perf, perforation.
AR, Anterior resection. APR, Abdominoperineal resection. CRM, circumferential resection margin.
scRT, short-course radiotherapy. scRT-delay, scRT with delay to surgery.
CRT, chemoradiotherapy. TNT, total neoadjuvant therapy. FU, fluoropyrimidine. [1]Pearson's Chi-squared test.

Table S3a. DM rates at 5 years after surgery grouped according to AJCC/pTN stage and RF

	RF	n	**Overall** % (95% CI, %)	n	**Surgery only** % (95% CI, %)	n	**scRT** % (95% CI, %)	n	**scRT-delay** % (95% CI, %)	n	**CRT/TNT** % (95% CI, %)
**Stage II**											
pT3ab	0	912	11 (9, 13)	406	9 (6, 12)	271	8 (4, 11)	101	21 (12, 30)	118	16 (9, 23)
	1	599	13 (10, 16)	167	10 (5, 16)	206	8 (4, 12)	88	20 (10, 29)	130	19 (12, 26)
	≥2	345	23 (18, 28)	79	23 (11, 34)	121	20 (12, 27)	61	24 (11, 36)	79	26 (15, 36)
pT3cd	0	223	10 (5, 14)	64	10 (2, 18)	69	7 (0, 13)	30	8 (0, 18)	55	12 (2, 20)
	1	200	17 (11, 22)	46	22 (8, 34)	58	9 (1, 17)	26	31 (9, 48)	63	15 (5, 24)
	≥2	185	35 (27, 43)	30	43 (21, 59)	69	27 (15, 38)	28	65 (33, 82)	53	33 (18, 46)
pT4	0	93	13 (5, 21)	29	12 (0, 24)	8	40 (0, 67)	20	31 (0, 58)	34	3 (0, 8)
	1	70	23 (12, 33)	21	32 (7, 50)	7	57 (0, 82)	10	26 (0, 52)	31	10 (0, 20)
	≥2	108	31 (21, 39)	27	34 (11, 51)	14	21 (0, 40)	21	45 (18, 63)	37	23 (8, 36)

	RF	n	**Total** % (95% CI, %)	n	**No Adjuvant Tx** % (95% CI, %)	n	**Adjuvant Tx** % (95% CI, %)	n	**Surgery only & No adjuvant** % (95% CI, %)	n	**Surgery only & Adjuvant Tx** % (95% CI, %)
**Stage II**											
pT3ab	0	912	11 (9, 13)	875	10 (8, 13)	37	22 (7, 35)	395	9 (6, 12)	11	27 (0, 49)
	1	599	13 (10, 16)	528	13 (10, 16)	71	14 (6, 22)	147	12 (6, 18)	20	0 (0, 0)
	≥2	345	23 (18, 28)	270	24 (18, 29)	75	21 (11, 30)	62	23 (9, 35)	17	24 (0, 41)
pT3cd	0	223	10 (5, 14)	208	9 (5, 13)	15	22 (0, 41)	63	9 (1, 16)	1	0 (0, 0)
	1	200	17 (11, 22)	166	15 (9, 21)	34	24 (8, 37)	42	19 (5, 31)	4	50 (0, 81)
	≥2	185	35 (27, 43)	121	34 (24, 43)	64	36 (23, 48)	20	25 (3, 42)	10	75 (20, 92)
pT4	0	93	13 (5, 21)	69	12 (3, 20)	24	15 (0, 29)	20	18 (0, 34)	9	0 (0, 0)
	1	70	23 (12, 33)	42	18 (5, 30)	28	29 (10, 44)	14	15 (0, 33)	7	62 (0, 86)
	≥2	108	31 (21, 39)	77	28 (17, 38)	31	37 (17, 52)	20	35 (7, 55)	7	29 (0, 55)

	RF	n	**Overall** % (95% CI, %)	n	**Surgery only** % (95% CI, %)	n	**scRT** % (95% CI, %)	n	**scRT-delay** % (95% CI, %)	n	**CRT/TNT** % (95% CI, %)
**Stage III (pN1)**											
T0	0	14	7 (0, 20)			1	0 (0, 0)	3	0 (0, 0)	8	13 (0, 33)
	1	17	31 (4, 50)			4	0 (0, 0)	2	0 (0, 0)	10	54 (7, 78)
	≥2	7	29 (0, 55)							7	29 (0, 55)
pT1-2	0	209	14 (9, 19)	105	12 (5, 19)	57	15 (5, 24)	25	13 (0, 26)	22	23 (3, 39)
	1	216	12 (7, 17)	86	9 (2, 16)	73	16 (6, 24)	22	20 (0, 39)	33	6 (0, 14)
	≥2	123	24 (15, 31)	46	17 (5, 27)	51	23 (10, 35)	9	37 (0, 62)	17	36 (8, 56)
pT3ab	0	245	13 (8, 17)	104	14 (6, 21)	69	9 (2, 16)	39	11 (0, 21)	32	16 (2, 29)
	1	226	22 (16, 27)	87	17 (8, 24)	82	25 (14, 35)	22	37 (10, 56)	32	19 (4, 31)
	≥2	245	39 (32, 45)	49	34 (16, 48)	102	31 (21, 40)	39	51 (29, 66)	53	53 (36, 66)
pT3cd	0	83	24 (14, 33)	31	32 (10, 49)	25	8 (0, 19)	9	60 (6, 83)	17	18 (0, 34)
	1	127	31 (22, 39)	29	22 (5, 37)	49	28 (14, 40)	13	38 (0, 62)	33	40 (20, 54)
	≥2	180	48 (38, 56)	47	40 (20, 54)	56	42 (27, 55)	31	57 (25, 76)	43	62 (41, 75)
pT4	0	26	34 (11, 51)	12	25 (0, 46)	6	44 (0, 77)	4	25 (0, 57)	3	67 (0, 93)
	1	41	42 (23, 57)	14	24 (0, 44)	6	40 (0, 71)	9	70 (12, 90)	11	52 (0, 78)
	≥2	98	55 (42, 65)	40	57 (36, 71)	14	74 (19, 92)	8	0 (0, 0)	35	54 (33, 69)

	RF	n	**Total** % (95% CI, %)	n	**No Adjuvant Tx** % (95% CI, %)	n	**Adjuvant Tx** % (95% CI, %)	n	**Surgery only & No adjuvant** % (95% CI, %)	n	**Surgery only & Adjuvant Tx** % (95% CI, %)
**Stage III (pN1)**											
T0	0	14	7 (0, 20)	10	0 (0, 0)	4	25 (0, 57)				
	1	17	31 (4, 50)	10	21 (0, 44)	7	43 (0, 70)				
	≥2	7	29 (0, 55)	3	33 (0, 70)	4	25 (0, 57)				
pT1-2	0	209	14 (9, 19)	101	21 (11, 29)	108	9 (3, 14)	49	19 (5, 30)	56	7 (0, 14)
	1	216	12 (7, 17)	106	17 (8, 24)	110	9 (3, 14)	35	16 (2, 29)	51	5 (0, 11)
	≥2	123	24 (15, 31)	67	27 (15, 38)	56	20 (9, 30)	29	27 (8, 43)	17	0 (0, 0)
pT3ab	0	245	13 (8, 17)	145	16 (9, 22)	100	8 (3, 14)	56	23 (9, 35)	48	4 (0, 10)
	1	226	22 (16, 27)	122	23 (15, 31)	104	20 (11, 27)	50	23 (10, 35)	37	8 (0, 17)
	≥2	245	39 (32, 45)	134	43 (32, 51)	111	35 (25, 44)	25	24 (0, 43)	24	42 (16, 60)
pT3cd	0	83	24 (14, 33)	45	22 (8, 34)	38	26 (10, 40)	17	22 (0, 41)	14	43 (4, 66)
	1	127	31 (22, 39)	68	33 (20, 44)	59	28 (15, 39)	17	28 (0, 49)	12	17 (0, 35)
	≥2	180	48 (38, 56)	93	53 (39, 64)	87	43 (30, 53)	27	48 (16, 68)	20	28 (3, 46)
pT4	0	26	34 (11, 51)	13	49 (11, 70)	13	19 (0, 40)	7	29 (0, 55)	5	20 (0, 48)
	1	41	42 (23, 57)	25	48 (20, 66)	16	34 (4, 54)	5	50 (0, 81)	9	11 (0, 29)
	≥2	98	55 (42, 65)	52	52 (35, 65)	46	57 (37, 70)	23	57 (28, 74)	17	52 (19, 72)

	RF	n	**Overall** % (95% CI, %)	n	**Surgery only** % (95% CI, %)	n	**scRT** % (95% CI, %)	n	**scRT-delay** % (95% CI, %)	n	**CRT/TNT** % (95% CI, %)
**Stage III (pN2)**											
T0	0	1	0 (0, 0)							1	0 (0, 0)
	1	1	0 (0, 0)							1	0 (0, 0)
	≥2	1	0 (0, 0)							1	0 (0, 0)
pT1-2	0	39	13 (2, 23)	26	8 (0, 18)	10	20 (0, 41)	0	0 (0, 0)	2	50 (0, 87)
	1	64	28 (14, 39)	12	0 (0, 0)	31	19 (2, 33)	10	0 (0, 0)	9	42 (0, 69)
	≥2	47	43 (26, 56)	15	47 (14, 67)	21	34 (10, 52)	3	0 (0, 0)	6	50 (0, 78)
pT3ab	0	83	27 (16, 36)	29	29 (7, 46)	34	26 (9, 40)	7	17 (0, 42)	12	34 (0, 57)
	1	93	30 (19, 39)	35	26 (8, 41)	31	30 (11, 44)	12	34 (0, 57)	12	42 (6, 64)
	≥2	129	52 (41, 60)	28	60 (32, 76)	50	49 (31, 62)	22	53 (23, 71)	20	56 (24, 74)
pT3cd	0	49	33 (17, 45)	14	21 (0, 40)	16	21 (0, 40)	6	40 (0, 71)	11	64 (21, 83)
	1	70	47 (33, 58)	22	55 (23, 74)	34	37 (18, 52)	6	0 (0, 0)	5	40 (0, 71)
	≥2	179	54 (45, 61)	47	49 (29, 63)	67	51 (36, 62)	23	49 (13, 70)	35	63 (41, 77)
pT4	0	12	33 (1, 55)	5	20 (0, 48)	4	25 (0, 57)	1	0 (0, 0)	0	0 (0, 0)
	1	36	45 (25, 60)	12	57 (12, 79)	10	30 (0, 53)	4	50 (0, 81)	8	40 (0, 67)
	≥2	75	48 (34, 59)	19	38 (8, 59)	28	45 (22, 61)	11	0 (0, 0)	12	57 (9, 80)

	RF	n	**Total** % (95% CI, %)	n	**No Adjuvant Tx** % (95% CI, %)	n	**Adjuvant Tx** % (95% CI, %)	n	**Surgery only & No adjuvant** % (95% CI, %)	n	**Surgery only & Adjuvant Tx** % (95% CI, %)
**Stage III (pN2)**											
T0	0	1	0 (0, 0)			1	0 (0, 0)				
	1	1	0 (0, 0)			1	0 (0, 0)				
	≥2	1	0 (0, 0)	1	0 (0, 0)						
pT1-2	0	39	13 (2, 23)	12	0 (0, 0)	27	19 (2, 32)	9	0 (0, 0)	17	12 (0, 26)
	1	64	28 (14, 39)	21	42 (8, 64)	43	22 (8, 34)	3	0 (0, 0)	9	0 (0, 0)
	≥2	47	43 (26, 56)	19	47 (19, 66)	28	39 (16, 56)	10	50 (7, 73)	5	40 (0, 71)
pT3ab	0	83	27 (16, 36)	36	31 (11, 47)	47	24 (11, 36)	12	69 (0, 93)	17	12 (0, 27)
	1	93	30 (19, 39)	33	51 (28, 67)	60	19 (8, 28)	12	52 (1, 77)	23	14 (0, 28)
	≥2	129	52 (41, 60)	50	65 (43, 78)	79	45 (32, 56)	8	0 (0, 0)	20	56 (27, 73)
pT3cd	0	49	33 (17, 45)	19	34 (7, 53)	30	32 (12, 47)	7	43 (0, 70)	7	0 (0, 0)
	1	70	47 (33, 58)	24	42 (13, 61)	46	49 (32, 62)	10	0 (0, 0)	12	50 (12, 72)
	≥2	179	54 (45, 61)	72	58 (39, 71)	107	53 (42, 62)	22	52 (16, 72)	25	47 (22, 64)
pT4	0	12	33 (1, 55)	5	60 (0, 86)	7	14 (0, 37)	3	33 (0, 70)	2	0 (0, 0)
	1	36	45 (25, 60)	12	37 (0, 61)	24	48 (22, 65)	3	0 (0, 0)	9	56 (8, 79)
	≥2	75	48 (34, 59)	33	60 (36, 75)	42	40 (20, 54)	12	50 (5, 74)	7	20 (0, 48)

Risk factors (RF) for DM: perineural invasion, vascular invasion, tumour deposit, lymph node yield, and tumour level 0-5 cm.
AJCC, American Joint Committee on Cancer. DM, distant metastatis. RF, risk factors. Tx, treatment. CI, confidence interval.
N1, 1-3 pathological lymph nodes. N2, 4 or more pathological lymph nodes.
scRT, short-course radiotherapy. scRT-delay, scRT with delay to surgery. CRT, chemoradiotherapy. TNT, total neoadjuvat therapy

Table S3b. LRR rates at 5 years after surgery grouped according to AJCC stage and RF

		**Overall**		**Surgery only**		**scRT**		**scRT-delay**		**CRT/TNT**	
Stage	RF	n	% (95% CI, %)	n	% (95% CI, %)	n	% (95% CI, %)	n	% (95% CI, %)	n	% (95% CI, %)
0	0	61	0 (0, 0)			2	0 (0, 0)	18	0 (0, 0)	39	0 (0, 0)
	1	204	2 (0, 5)			8	0 (0, 0)	62	0 (0, 0)	133	3 (0, 7)
	≥2	17	0 (0, 0)			1	0 (0, 0)	3	0 (0, 0)	12	0 (0, 0)
I	0	655	2 (1, 3)	476	1 (0, 3)	87	1 (0, 4)	41	5 (0, 12)	46	0 (0, 0)
	1	2064	2 (1, 2)	803	2 (1, 3)	669	1 (0, 2)	272	3 (1, 5)	302	1 (0, 2)
	≥2	293	3 (1, 5)	129	4 (0, 7)	97	1 (0, 3)	31	3 (0, 9)	34	4 (0, 11)
II	0	663	1 (0, 2)	376	2 (1, 3)	136	0 (0, 0)	57	0 (0, 0)	80	0 (0, 0)
	1	1551	3 (2, 4)	408	4 (2, 6)	540	2 (1, 3)	213	3 (0, 7)	360	5 (2, 7)
	≥2	862	5 (3, 6)	206	7 (3, 11)	272	2 (0, 4)	137	9 (3, 15)	223	5 (2, 7)
III	0	424	2 (0, 3)	255	2 (0, 3)	85	1 (0, 3)	34	4 (0, 11)	48	2 (0, 6)
	1	1230	3 (2, 4)	388	2 (1, 4)	452	3 (1, 5)	150	4 (1, 8)	222	4 (2, 7)
	≥2	1367	9 (8, 11)	363	14 (9, 18)	512	5 (3, 7)	170	8 (3, 13)	300	13 (8, 17)

		**Overall**		**No Adjuvant Tx**		**Adjuvant Tx**		**Surgery only & No adjuvant Tx**		**Surgery only & Adjuvant Tx**	
Stage	RF	n	% (95% CI, %)	n	% (95% CI, %)	n	% (95% CI, %)	n	% (95% CI, %)	n	% (95% CI, %)
0	0	61	0 (0, 0)	49	0 (0, 0)	12	0 (0, 0)				
	1	204	2 (0, 5)	183	2 (0, 4)	21	5 (0, 14)				
	≥2	17	0 (0, 0)	11	0 (0, 0)	6	0 (0, 0)				
I	0	655	2 (1, 3)	639	1 (0, 2)	16	6 (0, 17)	471	1 (0, 2)	5	20 (0, 48)
	1	2064	2 (1, 2)	1983	1 (1, 2)	81	3 (0, 6)	787	2 (1, 3)	16	0 (0, 0)
	≥2	293	3 (1, 5)	244	3 (1, 5)	49	2 (0, 7)	110	3 (0, 7)	19	6 (0, 17)
II	0	663	1 (0, 2)	625	1 (0, 2)	38	0 (0, 0)	367	2 (1, 4)	9	0 (0, 0)
	1	1551	3 (2, 4)	1376	3 (2, 4)	175	5 (1, 8)	367	3 (1, 5)	41	7 (0, 15)
	≥2	862	5 (3, 6)	629	5 (3, 7)	233	4 (1, 6)	156	8 (3, 13)	50	4 (0, 9)
III	0	424	2 (0, 3)	191	2 (0, 4)	233	1 (0, 3)	107	4 (0, 8)	148	0 (0, 0)
	1	1230	3 (2, 4)	626	4 (2, 6)	604	3 (1, 4)	196	3 (0, 6)	192	1 (0, 3)
	≥2	1367	9 (8, 11)	637	11 (8, 14)	730	8 (6, 10)	194	17 (10, 24)	169	10 (5, 15)

Risk factors (RF) for LRR: (y)pT4, tumour deposit, CRM, perforation near tumour, vascular invasion, and tumour levels 0-5 cm and 6-10 cm.

LRR, locoregional recurrence. AJCC, American Joint Committee on Cancer. RF, Risk factor. Tx, treatment. CI, confidence interval. CRM, circumferential resection margin. scRT, short-course radiotherapy. scRT-delay, scRT with delay to surgery. CRT, chemoradiotherapy. TNT, total neoadjuvat therapy

Table S4. Characteristics of treatment groups

Characteristic	Surgery only, N = 3415<br>N (% column)	scRT, N = 2786<br>N (% column)	scRT-delay, N = 1190<br>N (% column)	CRT, N = 1340<br>N (% column)	TNT, N = 334<br>N (% column)	p-value[1]
**Sex**						0.3
Female	1,406 (41%)	1,086 (39%)	485 (41%)	521 (39%)	123 (37%)	
Male	2,009 (59%)	1,700 (61%)	705 (59%)	819 (61%)	211 (63%)	
**Age,** years						**<0.001**
<75	2,120 (62%)	2,080 (75%)	639 (54%)	1,196 (89%)	307 (92%)	
≥75	1,295 (38%)	706 (25%)	551 (46%)	143 (11%)	27 (8%)	
**ASA**						**<0.001**
1	544 (16%)	608 (22%)	127 (11%)	309 (23%)	65 (19%)	
2	1,846 (54%)	1,637 (59%)	620 (52%)	796 (59%)	205 (61%)	
3	934 (27%)	486 (17%)	399 (34%)	219 (16%)	55 (16%)	
4	49 (1%)	20 (1%)	31 (3%)	2 (0%)	1 (0%)	
Unknown	42 (1%)	35 (1%)	13 (1%)	14 (1%)	8 (2%)	
**Tumour Level (cm)**						**<0.001**
11-15	1,612 (47%)	494 (18%)	233 (20%)	235 (18%)	80 (24%)	
6-10	1,315 (39%)	1,304 (47%)	493 (41%)	551 (41%)	132 (40%)	
0-5	451 (13%)	959 (34%)	457 (38%)	538 (40%)	119 (36%)	
Unknown	37 (1%)	29 (1%)	7 (1%)	16 (1%)	3 (1%)	
**Surgery**						**<0.001**
AR	2,154 (63%)	1,338 (48%)	431 (36%)	562 (42%)	170 (51%)	
APR	736 (22%)	1,233 (44%)	595 (50%)	687 (51%)	140 (42%)	
Hartmann	525 (15%)	215 (8%)	164 (14%)	91 (7%)	24 (7%)	
**AJCC Stage**						**<0.001**
I	1,408 (41%)	824 (30%)	344 (29%)	304 (23%)	61 (18%)	
0	0 (0%)	10 (0%)	83 (7%)	120 (9%)	50 (15%)	
II	990 (29%)	925 (33%)	402 (34%)	489 (36%)	136 (41%)	
III	1,006 (29%)	1,023 (37%)	353 (30%)	421 (31%)	82 (25%)	
Unknown	11 (0%)	4 (0%)	8 (1%)	6 (0%)	5 (1%)	
**pT**						**<0.001**
T1	510 (15%)	140 (5%)	81 (7%)	75 (6%)	15 (4%)	
T0	1 (0%)	16 (1%)	89 (7%)	142 (11%)	55 (16%)	
T2	1,188 (35%)	924 (33%)	332 (28%)	301 (22%)	58 (17%)	
T3ab	984 (29%)	940 (34%)	389 (33%)	356 (27%)	102 (31%)	
T3cd	330 (10%)	437 (16%)	172 (14%)	242 (18%)	52 (16%)	
T3 unknown	208 (6%)	230 (8%)	34 (3%)	92 (7%)	11 (3%)	
T4a	112 (3%)	59 (2%)	29 (2%)	35 (3%)	12 (4%)	
T4b	51 (1%)	22 (1%)	52 (4%)	83 (6%)	19 (6%)	
T4 unknown	16 (0%)	15 (1%)	6 (1%)	7 (1%)	3 (1%)	
Unknown	15 (0%)	3 (0%)	6 (1%)	7 (1%)	7 (2%)	
**pN**						**<0.001**
N0	2,353 (69%)	1,737 (62%)	825 (69%)	895 (67%)	249 (75%)	
N1a	399 (12%)	321 (12%)	122 (10%)	153 (11%)	32 (10%)	
N1b	309 (9%)	320 (11%)	118 (10%)	135 (10%)	29 (9%)	
N2a	175 (5%)	206 (7%)	60 (5%)	76 (6%)	14 (4%)	
N2b	123 (4%)	176 (6%)	53 (4%)	57 (4%)	7 (2%)	
Unknown	56 (2%)	26 (1%)	12 (1%)	24 (2%)	3 (1%)	
**Lymph Node Yield**						**<0.001**
≥12	2,986 (87%)	2,410 (87%)	1,013 (85%)	1,085 (81%)	254 (76%)	
<12	385 (11%)	359 (13%)	166 (14%)	244 (18%)	79 (24%)	
Unknown	44 (1%)	17 (1%)	11 (1%)	11 (1%)	1 (0%)	
**Tumor Deposit**						**<0.001**
No	2,824 (83%)	2,126 (76%)	966 (81%)	1,062 (79%)	273 (82%)	
Yes	292 (9%)	385 (14%)	156 (13%)	210 (16%)	45 (13%)	
Unknown	299 (9%)	275 (10%)	68 (6%)	68 (5%)	16 (5%)	
**Perforation**						0.6
No perforation	3,244 (95%)	2,669 (96%)	1,128 (95%)	1,273 (95%)	320 (96%)	
Perf not near tumor	68 (2%)	52 (2%)	31 (3%)	29 (2%)	6 (2%)	
Perf near tumor	66 (2%)	43 (2%)	23 (2%)	25 (2%)	8 (2%)	
Unknown	37 (1%)	22 (1%)	8 (1%)	13 (1%)	0 (0%)	
**Differentiation**						**<0.001**
High/moderate	2,885 (84%)	2,133 (77%)	924 (78%)	914 (68%)	225 (67%)	
Poor	379 (11%)	460 (17%)	121 (10%)	140 (10%)	39 (12%)	
Unknown	151 (4%)	193 (7%)	145 (12%)	286 (21%)	70 (21%)	
**CRM (mm)**						**<0.001**
≥ 2	2,935 (86%)	2,457 (88%)	964 (81%)	1,021 (76%)	241 (72%)	
1.1-1.9	265 (8%)	155 (6%)	130 (11%)	183 (14%)	68 (20%)	
≤1	197 (6%)	170 (6%)	87 (7%)	133 (10%)	21 (6%)	
**Mucinous**						**<0.001**
No	3,003 (88%)	2,422 (87%)	1,037 (87%)	1,108 (83%)	272 (81%)	
Yes	298 (9%)	265 (10%)	118 (10%)	158 (12%)	47 (14%)	
Unknown	114 (3%)	99 (4%)	35 (3%)	74 (6%)	15 (4%)	
**Vascular Invasion**						**<0.001**
No	2,542 (74%)	2,020 (73%)	911 (77%)	1,053 (79%)	246 (74%)	
Yes	807 (24%)	723 (26%)	236 (20%)	254 (19%)	65 (19%)	
Unknown	66 (2%)	43 (2%)	43 (4%)	33 (2%)	23 (7%)	
**Perineural Invasion**						**<0.001**
No	2,873 (84%)	2,131 (76%)	930 (78%)	1,062 (79%)	251 (75%)	
Yes	420 (12%)	544 (20%)	214 (18%)	236 (18%)	60 (18%)	
Unknown	122 (4%)	111 (4%)	46 (4%)	42 (3%)	23 (7%)	
**Adjuvant treatment**						**<0.001**
None	2,766 (81%)	2,032 (73%)	1,051 (88%)	831 (62%)	334 (100%)	
Only FU	373 (11%)	400 (14%)	93 (8%)	226 (17%)	0 (0%)	
FU + Oxaliplatin	276 (8%)	354 (13%)	46 (4%)	283 (21%)	0 (0%)	
**Locoregional recurrence**						**<0.001**
No	3,309 (97%)	2,730 (98%)	1,148 (96%)	1,290 (96%)	320 (96%)	
Yes	106 (3%)	56 (2%)	42 (4%)	50 (4%)	14 (4%)	
**Distant metastasis**						**<0.001**
No	2,992 (88%)	2,323 (83%)	959 (81%)	1,054 (79%)	265 (79%)	
Yes	423 (12%)	463 (17%)	231 (19%)	286 (21%)	69 (21%)	

ASA, American Society of Anethesiologists Physical Status. AJCC, American Joint Committee on Cancer.
AR, anterior resection. APR, abdominoperineal resection. CRM, circumferential resection margin. Perf, perforation.
scRT, short-course radiotherapy. scRT-delay, scRT with delay to surgery
CRT, chemoradiotherapy. TNT, total neoadjuvant therapy. FU, fluoropyrimidine. [1]Pearson's Chi-squared test.

FIGURE S1.

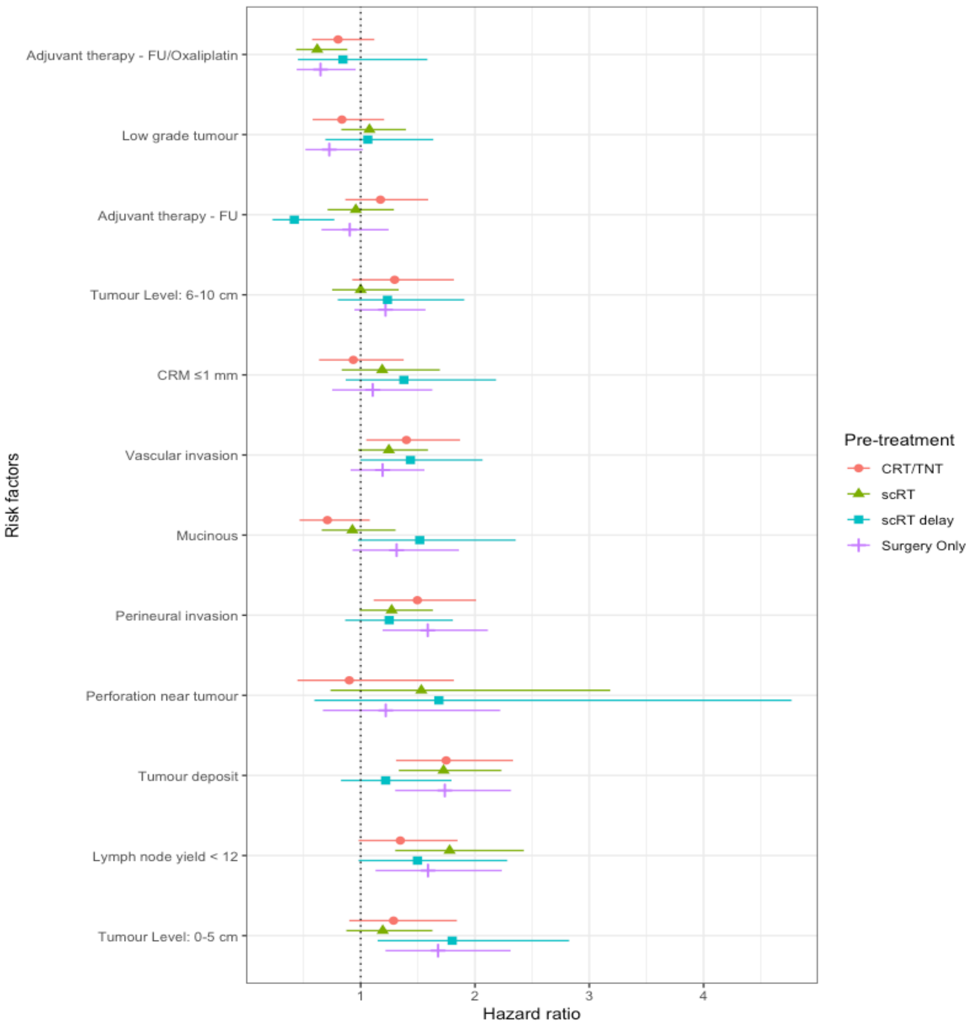
Adjuvant therapy - FU/Oxaliplatin
Low grade tumour
Adjuvant therapy - FU
Tumour Level: 6-10 cm
CRM ≤1 mm
Vascular invasion
Mucinous
Perineural invasion
Perforation near tumour
Tumour deposit
Lymph node yield < 12
Tumour Level: 0-5 cm
Risk factors
1
2
3
4
Hazard ratio
Pre-treatment
CRT/TNT
scRT
scRT delay
Surgery Only

Figure S1. Forest plot depicting HR for DM stratified by pre-treatment.
HR, Hazard ratio, DM, distant metastatis. CRM, circumferential resection margin. scRT, short-course radiotherapy. scRT-delay, scRT with delay to surgery. CRT, chemoradiotherapy. TNT, total neoadjuvant therapy. FU, fluoropyrimidine
